# Dynamical stability by spin transfer in nearly isotropic magnets

**DOI:** 10.1038/s41563-026-02510-z

**Published:** 2026-03-04

**Authors:** Hidekazu Kurebayashi, Joseph Barker, Takumi Yamazaki, Varun K. Kushwaha, Kilian D. Stenning, Harry Youel, Xueyao Hou, Troy Dion, Daniel Prestwood, Gerrit E. W. Bauer, Kei Yamamoto, Takeshi Seki

**Affiliations:** 1 London Centre for Nanotechnology, University College London, London, UK; 2 Department of Electronic and Electrical Engineering, University College London, London, UK; 3 Center for Science and Innovation in Spintronics, Tohoku University, Sendai, Japan; 4 Institute for Materials Research, Tohoku University, Sendai, Japan; 5 WPI Advanced Institute for Materials Research, Tohoku University, Sendai, Japan; 6 School of Physics and Astronomy, University of Leeds, Leeds, UK; 7 Blackett Laboratory, Imperial College London, London, UK; 8 Department of Physics and Astronomy, University College London, London, UK; 9 Kavli Institute for Theoretical Sciences, University of the Chinese Academy of Sciences, Beijing, China; 10 Advanced Science Research Center, Japan Atomic Energy Agency, Tokai, Japan

**Keywords:** Spintronics, Spintronics

## Abstract

Spin transfer torques (STTs) control magnetization by electric currents, enabling a range of nano-scale spintronic applications. They can destabilize the equilibrium magnetization state by counteracting magnetic relaxation. Here we maximize the STT effect through a dedicated growth-annealing protocol for CoFeB thin films, such that magnetic anisotropies originating from the interface and shape almost cancel each other. The nearly isotropic magnets enable low-current dynamical stabilization of the magnetization in the direction opposite to an applied magnetic field, thereby realizing a spintronic analogue of the Kapitza pendulum. In an intermediate current regime, the STT drives large magnetization vector fluctuations that cover the entire Bloch sphere. The continuous variable associated with the stochastic magnetization direction may serve as a resource for probabilistic computing and neuromorphic hardware. Our results establish isotropic magnets as a platform to study as-yet-uncharted, far-from-equilibrium spin dynamics including anti-magnonics, with promising implications for unconventional computing paradigms.

## Abstract (web summary)

Spin torques generated via the spin-Hall effect in CoFeB/W/MgO are found to stabilize magnetization in a high-energy anti-parallel state relative to an applied magnetic field. This observation serves as a platform for studying far-from-equilibrium spin dynamics and holds promise for realizing unconventional computing paradigms.

## Main

**Fig. 1:**
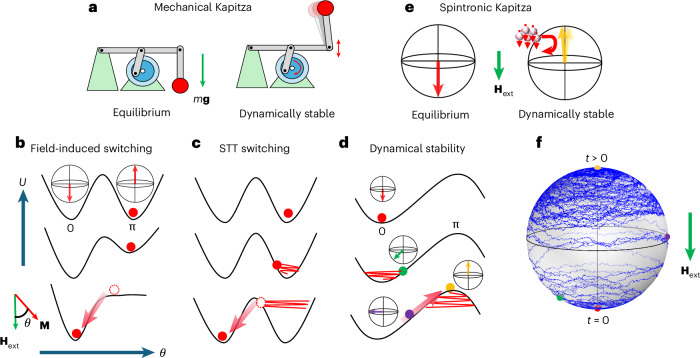
Dynamical stability of the spintronic Kapitza pendulum. **a**, Schematics of the mechanical Kapitza pendulum with and without an external drive. *m***g** represents the gravitational force on the bob. **b**–**d**, Schematics of different magnetization switching and stability processes illustrated within the angular dependence of magnetic energy (*U*): field-induced switching (**b**), STT switching (**c**) and dynamical stability (**d**). *θ* is defined by the relative angle between the external magnetic field (**H**_ext_) and magnetic moment (**M**). See below for the mechanism of each process. **e**, Analogy between the Kapitza pendulum and dynamical stabilization by spin transfer in an isotropic magnet. The magnetic field provides a minimum and maximum of potential energy as the gravitational field does, and the STT plays the role of dynamical driving that controls their stability. Note that the arrows piercing through the conduction electrons (solid spheres) represent their spin pointing opposite to their magnetic moment. **f**, A typical solution of the stochastic LLG equation for a macrospin in an isotropic magnet. The initial condition is set at the south pole (red dot) and due to the anti-damping STT exceeding the Gilbert damping torque, the state precesses away from the field direction and settles around the inverted state (yellow dot). The coloured dots on the sphere have the corresponding potential energy values indicated in **d**.

A rigid pendulum whose pivot is forced to vibrate up and down, known by the name of Kapitza^1^, has inspired generations of physicists^2–4^. Under fast actuation, the bob appears to defy gravity by staying close to the upright position (Fig. 1a), demonstrating so-called dynamical stability^5^. It represents a real-world example of a controllable nonlinear dynamical system that exhibits a range of steady states in the long term, called attractors^6^, such as complex periodic orbits^7^, as well as chaotic motion^8^. The fixed distance between the bob and the pivot drastically simplifies the equation of motion but preserves the nonlinearity that causes the counter-intuitive dynamics.

Magnetic order shares the rigidity of a pendulum because the high exchange energy cost of modulating the saturation magnetization *M*_s_ constrains the vector **M** to the Bloch sphere with radius *M*_s_. The Landau–Lifshitz–Gilbert (LLG) torque equation, a cousin of Newton’s second law, relates the rate of change of the spin angular momentum density −*γ*^−1^d**M**/d*t* to the sum of various torques per volume **M** × *δ*_**M**_*U* + **τ** that act on the magnetization. Here *γ* > 0 is the modulus of the gyromagnetic ratio, *U* is the magnetic free energy, *δ*_**M**_ denotes the functional derivative with respect to **M** and **τ** includes the viscous Gilbert damping^9^ and other torques that do not conserve *U*. Most studies focus on switching the magnetic order between the north and south poles of the Bloch sphere, which are the energy minima in the presence of a uniaxial anisotropy. This bistability is employed in magnetic memory applications^10^ in which the two poles of a small magnetic medium represent a single bit that can be read out electrically by the celebrated giant/tunnelling magnetoresistance (MR)^11–14^. The barrier between the energy minima is sufficiently higher than the thermal energy to retain the information safely^15–17^.

A static magnetic field tilts the free energy double-well and favours one of the two minima into which the magnetization settles in equilibrium (Fig. 1b). The magnetization can be flipped by reversing the magnetic field but also electrically using current-induced spin transfer from spin-polarized conduction electrons^18,19^ that contributes to **τ** without affecting *U* (ref. ^20^). This effect, so-called spin transfer torques (STTs)^21–23^, may counteract the magnetic dissipation that stabilizes the magnetization against fluctuations around a free energy minimum^24^. When it overcomes the damping, the equilibrium becomes unstable, sending the system into motion, similar to what the oscillating pivot does to a rigid pendulum. In the presence of a large magnetic anisotropy, the switching is between the potential minima and non-volatile^25–27^ as illustrated in Fig. 1c. Two decades ago, a volatile current-driven switching behaviour was observed once the applied magnetic field exceeds the anisotropy field^28^. It was subsequently identified to be a dynamical stabilization at a potential maximum^29–31^ (Fig. 1d), reminiscent of the Kapitza pendulum. The physical mechanism is different, however, in the dissipative nature of STT contrasted to the effective conservative potential description of the driven pendulum where the inverted state is a local minimum^1^. The high field and current density required for the dynamical stability in the hard magnetic layers in a nanopillar structure hindered further investigation of this regime.

In this study, we employ magnetic films with vanishingly small magnetic anisotropy as a material platform to investigate dynamical stability and associated nonlinear phenomena. As a model system, we use prototypical MgO∣CoFeB∣W multilayers in which the demagnetizing field in the thin-film magnets favours CoFeB magnetization within the film plane but can be counter-balanced by an interface-induced perpendicular magnetic anisotropy^15–17^ (PMA) controlled by post-growth annealing (see Supplementary Note 1 for more details). We drive its nonlinear dynamics by the STT generated by the spin-orbit interaction in the tungsten layer (spin-orbit torque, SOT)^32–34^. Two independent electrical measurements confirm that the STT forces the magnetization into a steady state residing at the free energy maximum, contradicting the common wisdom that the attractor state under a large STT is an auto-oscillation, a particular kind of nonlinear periodic orbit that appears as uniform and localized spin-wave modes^35–40^. A vanishing anisotropy suppresses auto-oscillations and drastically reduces the critical current to drive the magnet into the nonlinear dynamical regime. Our analytical estimates and numerical simulations (for example, Fig. 1f) show that the probability distribution of the magnetization direction on the sphere can be controlled at will by the current and field. This whole Bloch sphere sampling might resource a continuous variable for probabilistic computation hardware, different from currently studied binary magnetic systems^41,42^. Our devices serve as a foundation for studying nonlinear dynamics of a complex many-body system that can be understood by a simple model.

## Minimizing the magnetic anisotropy

We grew multilayer stacks of MgO(3 nm)∣CoFeB(2 nm)∣W(3 nm) in an ultrahigh vacuum sputtering system on thermally oxidized Si at room temperature (Methods). As-grown films are easy-plane magnets due to strong demagnetizing fields of magnitude *M*_s_. The PMA from the CoFeB∣MgO interface^43,44^ tends to pull the magnetization out of plane which can be incorporated by an effective magnetization *M*_eff_ = *M*_s_ − 2*K*_⊥_/(*μ*_0_*M*_s_), where *K*_⊥_ is the perpendicular interface anisotropy constant and *μ*_0_ is the permeability of vacuum. By careful growth and post-annealing we tune the system to *M*_eff_ ≈ 0 as explained in Supplementary Note 1.

**Fig. 2:**
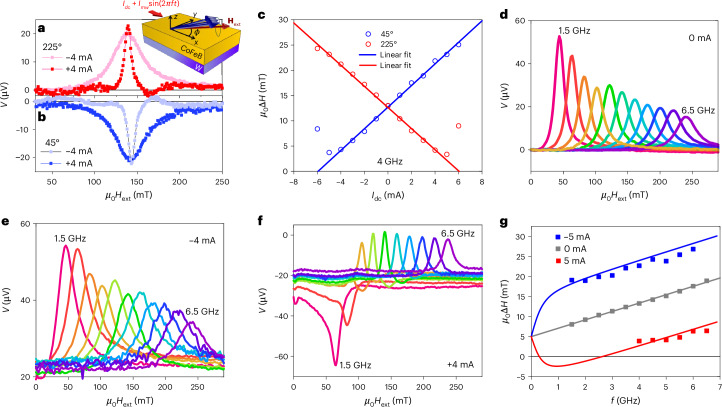
Current-induced magnetic damping probed by FMR. **a**,**b**, Field-swept FMR voltages measured for *ϕ* = 225^∘^ (**a**) and *ϕ* = 45^∘^ (**b**). The inset is a sketch of our device and the coordinate system. **c**, Linewidth extracted from FMR at 4 GHz with *ϕ* = 45^∘^ (blue) and *ϕ* = 225^∘^(red), for different *I*_dc_. The solid lines show the results of a linear fitting excluding points below −4 mA for 45^∘^ and above 4 mA for 225^∘^. **d**–**f**, Field-swept FMR voltages measured for various frequencies (0.5 GHz separations) and three values of *I*_dc_ at 0 mA (**d**), **−**4 mA (**e**) and 4 mA (**f**). Magnetic fields were applied along *ϕ* = 225^∘^. **g**, Linewidth as a function of frequency for different *I*_dc_, that is +5, 0 and −5 mA, represented by the red, grey and blue dot, respectively. The measurements were carried out at *ϕ* = 225^∘^. The grey line is a linear fitting of the 0 mA data. Both red and blue curves are calculated using equation (2).

In the coordinate system in the inset of Fig. 2a, the spin-Hall effect initiates the spin transfer by converting a charge current in the *x* direction into a spin current normal to the interface and polarized along the *y* direction^32,45^. The SOT arising from a d.c. current *I*_dc_ destabilizes the equilibrium **M**∥**H**_ext_ at a critical value *I*_c_ (refs. ^28,29,46^) that solves

1 $$-\beta {I}_{{\rm{c}}}\sin \phi =\alpha \left({H}_{\mathrm{ext}}+\frac{{M}_{\mathrm{eff}}}{2}\right)+\Delta H^{\prime}_{0},$$

where *α*, *H*_ext_ and *ϕ* are the Gilbert damping constant, the external magnetic field magnitude and the angle between the *x* axis and **H**_ext_ applied within the film plane, respectively. *β* is a phenomenological parameter proportional to the spin-Hall angle *θ*_SH_ (Methods) that characterizes the efficiency of the charge-to-spin conversion. In Supplementary Note 3, we explain that the current-induced self-torque in the CoFeB layer is negligibly small because electrons flow primarily in the W layer. Spatial variations of the material parameters require the introduction of a constant offset $\Delta H^{\prime}_{0}$. We can reduce the critical current by minimizing *M*_eff_, as expected.

## Current rectification in the inverted state

The dynamical stability of the anti-parallel state ${\bf{M}}={({M}_{x},{M}_{y},{M}_{z})}^{T}\parallel -{{\bf{H}}}_{\mathrm{ext}}$ at large negative currents *I*_dc_ can be detected by measuring the spin-Hall MR (SMR)^47,48^ in static and dynamical regimes. The angular dependence of the electric resistance reads $R={R}_{0}+\Delta {R}_{\mathrm{SMR}}(1-{M}_{y}^{2}/{M}_{\,{\rm{s}}}^{2})$ (ref. ^47^). See detailed experiments of SMR in Supplementary Note 3. Here *R*_0_ is the bulk resistance, whereas Δ*R*_SMR_ can be calculated by spin-diffusion theory with appropriate boundary conditions^48^ and/or fitted to the experiments. When a microwave current ${I}_{\mathrm{mw}}\cos \left(2{\rm{\pi }}ft\right)$ at frequency *f* modulates *I*_dc_, the induced ac SOT causes a small oscillation *M*_s_*δm*_*y*_ in *M*_*y*_ that, in turn, affects the electric resistance. The resulting rectified voltage *V* as a function of *f* and *H*_ext_ is monitored by the spin-torque ferromagnetic resonance (ST-FMR)^45,49,50^. According to Supplementary Note 4, the resonance field and current-dependent linewidth are given by ${H}_{\mathrm{res}}^{\pm }=\sqrt{4{{\rm{\pi }}}^{2}{f}^{2}/{(\gamma {\mu }_{0})}^{2}+{M}_{\mathrm{eff}}^{2}/4}\mp {M}_{\mathrm{eff}}/2$ and

2 $$\Delta {H}_{\pm }=\Delta {H}_{0}\pm \frac{2{\rm{\pi }}f}{\gamma {\mu }_{0}}\left(\alpha +\frac{\beta {I}_{\mathrm{dc}}\sin \phi }{{H}_{\mathrm{res}}^{\pm }\pm {M}_{\mathrm{eff}}/2}\right),$$

respectively, where the upper (lower) sign holds for the parallel (anti-parallel) state, and an inhomogeneous broadening Δ*H*_0_ is assumed independent of the magnetization direction. To the leading order, $V\approx -2\Delta {R}_{\mathrm{SMR}}{I}_{\mathrm{mw}}({M}_{y}/{M}_{{\rm{s}}})\overline{\delta {m}_{y}\cos \left(2{\rm{\pi }}ft\right)}$, where the overbar denotes time averaging over a period. As $\delta {m}_{y}\approx \beta {I}_{\mathrm{mw}}\cos (2{\rm{\pi }}ft)/\Delta {H}_{\pm }$ at ${H}_{{\rm{ext}}}={H}_{{\rm{res}}}^{\pm }$ and Δ*H*_±_ > 0 whenever **M**∥ ± **H**_ext_ is stable, *V* changes sign on magnetization reversal. With ${M}_{y}=\pm {M}_{{\rm{s}}}\sin \phi$, the *H*_ext_ dependence of *V* is given by

3 $$V=\mp \Delta {R}_{\mathrm{SMR}}\beta {I}_{\mathrm{mw}}^{2}\frac{2{\rm{\pi }}f/(\gamma {\mu }_{0})}{2{H}_{\mathrm{res}}^{\pm }\pm {M}_{\mathrm{eff}}}\frac{\Delta {H}_{\pm }{\cos }^{2}\phi \sin \phi }{{\left({H}_{\mathrm{ext}}-{H}_{\mathrm{res}}^{\pm }\right)}^{2}+\Delta {H}_{\pm }^{2}}.$$

The Lorentzian approximation (equation (3)) breaks down when ${H}_{\mathrm{ext}}\approx {H}_{\mathrm{res}}^{\pm }$ and Δ*H*_±_ ≈ 0 simultaneously (Supplementary Note 4).

## Dynamical stability

Figure 2 summarizes our ST-FMR experiments on devices fabricated from magnetic multilayers with vanishing anisotropies as shown in Supplementary Fig. 1f. The ferromagnetic resonance (FMR) traces for *ϕ* = 45^∘^ and 225^∘^ under applied currents *I*_dc_ = ± 4 mA at *f* = 4 GHz in Fig. 2a,b display clear linewidth changes^51^ as predicted by equation (2). The resonance fields *H*_res_ and linewidths Δ*H* extracted by a double Lorentzian fit (Methods) are plotted in Fig. 2c and confirm the linear relationship between Δ*H* and *I*_dc_ up to currents close to *I*_c_ defined by equation (1). At higher currents, the system becomes increasingly unstable, and Δ*H* increases again. Linear extrapolation to zero Δ*H* gives *I*_c_ ≈ 6 mA at *μ*_0_*H*_ext_ = *μ*_0_*H*_res_ ≈ 140 mT or a current density of 2 × 10^11^ A m^−2^, which is approximately one order of magnitude smaller than the typical values for the onset of auto-oscillation in anisotropic magnets^37,52^, due to the minimized *M*_eff_. The deviation from the linear relation near *I*_c_ reflects the aforementioned breakdown of equation (3).

The FMR lineshape for *ϕ* = 225^∘^ and *I*_dc_ of 0 and −4 mA in Fig. 2d,e affirms the expected modulation of Δ*H*, whereas fitting the field-frequency relation of the peak frequencies by the Kittel formula yields the residual easy-plane anisotropy of *μ*_0_*M*_eff_ = 37.3 mT, which is indeed much smaller than *μ*_0_*M*_s_ of CoFeB (~1.5 T)^53^. At *I*_dc_ = 4 mA in Fig. 2f, *V* changes its sign at low frequencies, which is strong evidence of the dynamical stabilization of the anti-parallel state **M**∥ − **H**_ext_ for *μ*_0_*H*_ext_ ≲ 100 mT. This also agrees with equation (1) where *I*_c_ = 4 mA for *ϕ* = 225^∘^ and *μ*_0_*H*_ext_ = 100 mT. The fit of the linear relation in Fig. 2c leads to *θ*_SH_ = 0.22, which is similar to published values^54^ and *β* = 2.6 × 10^6^ m^−1^. We confirm the sign reversal of the ST-FMR peaks for small *f* and *H*_ext_ in different samples as shown in Supplementary Fig. 10. Equation (2) offers a quantitative test of our interpretation. For $2{\rm{\pi }}f/\gamma \gg \left|{M}_{\mathrm{eff}}\right|$ and ${H}_{\mathrm{res}}^{+}+{M}_{\mathrm{eff}}/2\propto f$, Δ*H* becomes a linear function of *f* with a slope 2π*α*/*γμ*_0_ that does not depend on *I*_dc_. Figure 2g verifies this prediction, as *I*_dc_ mainly produces the Δ*H* offset of $2{\rm{\pi }}f\beta {I}_{\mathrm{dc}}\sin \phi /\sqrt{4{{\rm{\pi }}}^{2}{f}^{2}+{\gamma }^{2}{\mu }_{0}^{2}{M}_{\mathrm{eff}}^{2}/4}$ for 2π*f* ≫ *γμ*_0_*M*_eff_, in agreement with the curves that represent equation (2) for the upper signs with *α* = 0.053 and *μ*_0_Δ*H*_0_ = 5 mT.

## Electric resistance probes magnetic fluctuations

The thermal fluctuations of the magnetization angle affect its time-averaged properties. In particular, when the stability of both poles near the critical current weakens, the magnetization becomes susceptible to even small random torques. Because the time scales associated with the motion of conduction electrons (femtosecond) are much faster than those of magnetic fluctuations (nanosecond), the SMR probes the expectation value of ${M}_{y}^{2}$, that is, $R={R}_{0}+\Delta {R}_{\mathrm{SMR}}(1-\langle {M}_{y}^{2}\rangle /{M}_{{\rm{s}}}^{2})$, in which the angled brackets denote temporal and spatial averaging^47^. Larger fluctuations of **M** imply smaller $\langle {M}_{y}^{2}\rangle$ and a higher resistance in the parallel state (see Supplementary Note 3 for more discussions in relation to unidirectional SMR^55,56^).

**Fig. 3:**
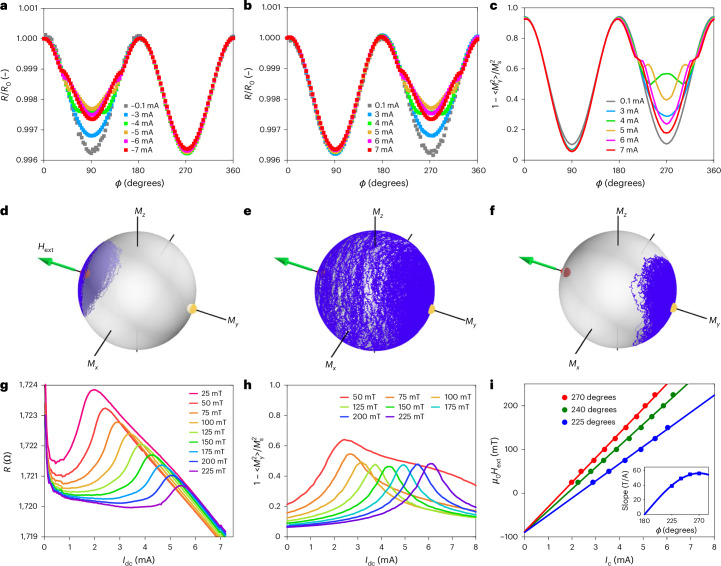
Electric resistance probes for magnetic fluctuations and the inverted states. **a**,**b**, Observed angular MR in the coordinate system of Fig. 2a for negative (**a**) and positive (**b**) currents, normalized by the resistance at *ϕ* = 0^∘^ for each current. **c**, $1-\langle {M}_{y}^{2}\rangle /{M}_{\,{\rm{s}}}^{2}$ calculated by the stochastic LLG equation in the macrospin approximation. **d**–**f**, Time-dependent trajectories of the macrospin moment calculated by the stochastic LLG equation for currents 0.1 mA (**d**), 4.1 mA (**e**) and 7 mA (**f**) and *ϕ* = 270^∘^. The field strength is 125 mT for **a**–**f**. **g**, Resistance versus d.c. current measurements for applied magnetic fields 25–200 mT along *ϕ* = 270^∘^. **h**, Calculated $1-\langle {M}_{y}^{2}\rangle /{M}_{{\rm{s}}}^{2}$ as a function of current and applied magnetic field. **i**, *I*_c_ extracted in **g** for different field strengths and angles (dots) and linear fits (lines). The inset shows the slope (dots) and a fit by $\sin \phi$ (curve).

Figure 3a,b shows the *ϕ* dependence of the MR for *μ*_0_*H*_ext_ = 150 mT and different *I*_dc_. Without fluctuations, $$R/{R}_{0}=1+\left(\Delta {R}_{\mathrm{SMR}}/{R}_{0}\right)$$$$(1-{\sin }^{2}\phi )$$, which agrees well for small applied currents *I*_dc_ = ± 0.1 mA. Joule heating cannot explain the asymmetric distortion of the MR around *ϕ* = 180^∘^ at larger $$\left|{I}_{\mathrm{dc}}\right|$$. A strong current dependence occurs only when the field direction and spin polarization are parallel, that is, when the STT destabilizes the equilibrium magnetization. Beyond currents of 4 and 5 mA, the fluctuations appear to decrease again.

We model the magnetization fluctuations by a stochastic LLG equation that includes the thermal noise in the effective magnetic field ***ξ*** that obeys the fluctuation–dissipation relation $$\langle {\xi }_{i}\left(t\right){\xi }_{j}\left(t{\prime} \right)\rangle$$$$={\delta }_{{ij}}\delta ({t-t}^{{{{\prime} }}})(2\alpha {k}_{{\rm{B}}}T)/(\gamma (1+{\alpha }^{2}){M}_{{\rm{s}}}{V}_{{\rm{a}}})$$, where *k*_B_ is the Boltzmann constant and *V*_a_ = 1 × 25 × 25 nm^3^ is a magnetic coherence volume^57^. The calculated $$1-\langle {M}_{y}^{2}\rangle /{M}_{{\rm{s}}}^{2}$$ as a function of *ϕ* for fixed *I*_dc_ in Fig. 3c reproduces the salient trends observed in Fig. 3b. **M**(*t*) for current values *I*_dc_ = 0.1, 4.1 and 7 mA and *ϕ* = 270^∘^ in Fig. 3d–f illustrate the build-up of the steady state probability distributions in Fig. 3c. For very small and large currents, at least at one of the poles the torques damp rather than amplify the thermal agitation. Close to the critical current (Fig. 3e), the magnetization vector explores the entire Bloch sphere with nearly constant probability density.

Equation (1) should hold near the resistance maxima in Fig. 3g that shift towards higher *I*_dc_ for larger *H*_ext_ but disappear when switching the field direction as shown in Supplementary Fig. 12a. At *μ*_0_*H*_ext_ = 150 mT the resistance peak height relative to lower *I*_dc_ is about 1–2 Ω or 0.1%, consistent with Fig. 3b at *ϕ* = 270^∘^. The peaks in the calculated $1-\langle {M}_{y}^{2}\rangle /{M}_{{\rm{s}}}^{2}$ in Fig. 3h correspond to a minimum in $\langle {M}_{y}^{2}\rangle /{M}_{{\rm{s}}}^{2}$ or maximum of the magnetic fluctuations at *I*_c_. The differences between theory and experiments (Fig. 3g,h) are partially caused by the incomplete theoretical account of heating effects, as well as the limitation of the macrospin model to describe spatial fluctuations in our extended film geometry. Equation (1) predicts a linear relationship between *H*_ext_ and *I*_c_ with a negative intercept. Figure 3i illustrates the linear field-current relationship observed at the resistance maximum. A fit to the *ϕ* dependence of the slope (Fig. 3i, inset, and Supplementary Fig. 11e) leads to the ratio *θ*_SH_/*α* = 3.7. Substituting *α* = 0.053 from the ST-FMR, *θ*_SH_ = 0.20 agrees well with *θ*_SH_ = 0.22 found above from an independent experiment on the same sample. Using equation (1) with the experimental parameters *α* and *M*_eff_ and the zero-current intercept in Fig. 3i, ${\mu }_{0}\Delta H^{\prime}_{0}=3.8$ mT, comparable to the inhomogeneous broadening *μ*_0_Δ*H*_0_ = 5.0 mT as deduced from the ST-FMR.

## Stochastic switching in nearly isotropic magnets

**Fig. 4:**
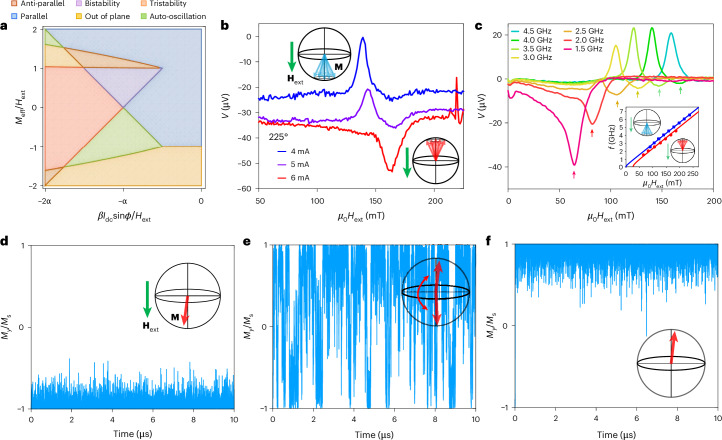
Stability diagram, bistability and probabilistic distribution for nearly isotropic magnets. **a**, Bifurcation diagram constructed from the stationary solutions of the LLG equation. **b**, ST-FMR for different *I*_dc_ close to the damping compensation condition. When a larger *I*_dc_ increases the anti-damping torques beyond the critical value, the magnetization precesses around the dynamically stable state at the north pole, generating an opposite d.c. voltage at resonance. **c**, The ST-FMR results highlight the current-induced magnetization reversal. The inset shows the FMR frequencies as functions of field for the two magnetization directions. **d**–**f**, Time-dependent traces of the normalized *M*_*y*_ component for small (**d**)/medium (**e**)/large (**f**) anti-damping torques. Here, *M*_*y*_ = −1 is along the magnetic field direction.

Both ST-FMR and MR measurements find a residual easy-plane anisotropy *M*_eff_ > 0 that, albeit small, causes an effect on the nonlinear dynamics that deserves a more detailed study. Figure 4a shows a bifurcation (or dynamical phase) diagram that analytically classifies the asymptotic final states of the deterministic LLG equation as a function of dimensionless parameters $\beta {I}_{{\rm{dc}}}\sin \phi /{H}_{{\rm{ext}}}$ and *M*_eff_/*H*_ext_ (see Supplementary Note 5 for more details). Since the Poincaré–Bendixon theorem^58^ excludes chaos on a sphere, the attractors in the absence of stable time-independent solutions must be finite-angle precessional states. The ‘auto-oscillations’ that occupy the regions shaded in green exist for *M*_eff_ > 0 only when the anisotropy and STT are very large. We identify a regime around the damping compensation $-\beta {I}_{{\rm{dc}}}\sin \phi /{H}_{{\rm{ext}}}=\alpha$, in which the inverted state is dynamically stabilized by a current that is smaller than the estimate by equation (1) for *M*_eff_ = 0. We may explain this deviation in terms of a dynamical bistability, also observed in the simulations in Fig. 3e in the form of a bimodal probability density at the critical current.

The experiments show clear signatures of this bistability. The ST-FMR voltages in Fig. 4b change sign with increasing *I*_dc_. The FMR mode around **M**∥**H**_ext_ at *μ*_0_*H*_ext_ ≈ 140 mT gradually disappears, and an inverted peak emerges at a higher field *μ*_0_*H*_ext_ ≈ 160 mT. This observation supports the prediction of different resonance fields ${H}_{{\rm{res}}}^{+}-{H}_{{\rm{res}}}^{-}=-{M}_{{\rm{eff}}}$ in equation (3). At *I*_dc_ = 5 mA, two peaks are observed. The one at *μ*_0_*H*_ext_ ≈ 140 mT for **M**∥**H**_ext_ is, according to equation (1), stable at higher fields. The inverted peak at *μ*_0_*H*_ext_ ≈ 160 > 140 mT, indicates the need for two independent stability criteria. Figure 4c also shows two peaks in ST-FMR voltages at different frequencies and a fixed current of *I*_dc_ = 4 mA for 100 mT ≲ *μ*_0_*H*_ext_ ≲ 170 mT. Theory predicts different resonance conditions for the parallel and inverted states $2{\rm{\pi }}{f}_{\mathrm{res}}^{\pm }=\gamma {\mu }_{0}\sqrt{{H}_{\mathrm{ext}}\left({H}_{\mathrm{ext}}\mp {M}_{\mathrm{eff}}\right)}$ for **M**∥ ± **H**_ext_ respectively, consistent with ${H}_{{\rm{res}}}$ in the inset of Fig. 4c, which allows us to estimate *μ*_0_*M*_eff_ ≈ 30 ± 5 mT.

Our discovery of dynamically stabilized states opens a vast and unexplored field of research. The numerical solutions of the stochastic LLG equation for *M*_eff_/*H*_ext_ = 0.31 in Fig. 4d–f show drastic changes of the probabilistic distribution when relaxing the constraint of a strong anisotropy. At an intermediate current *I*_dc_ = 4.0 mA, the magnetization visits all angles of the Bloch sphere with comparable probability. The characteristic dwell time around the poles in Fig. 4e is in the submicrosecond range, consistent with a semi-analytical estimate from the ‘first-passage time’ theory^59,60^ (Supplementary Note 7). It is predicted to be highly tunable through an exponential dependence on a Boltzmann factor *μ*_0_*M*_s_*H*_ext_*V*_a_/*k*_B_*T*, which can be probed by time-resolved optical or electric measurements. An analytical solution of the Fokker–Planck equation corresponding to the stochastic LLG for *M*_eff_ = 0 gives the same exponential dependence on *μ*_0_*M*_s_*H*_ext_*V*_a_/*k*_B_*T* of the MR versus *I*_dc_ (Supplementary Note 6). Supplementary Fig. 12, on the other hand, shows that the current dependence of the MR is remarkably robust across a wide range of temperature. We attribute the discrepancies to spatial fluctuations in the film beyond the macrospin model, such as sample inhomogeneities, domain formation or spin-wave instabilities of the anti-parallel state^61^. Furthermore, our understanding of the crystalline, compositional and thickness inhomogeneities and their effects on the critical currents is incomplete, as is our interpretation of $\Delta H^{\prime}_{{0}}$ in equation (1).

**Fig. 5:**
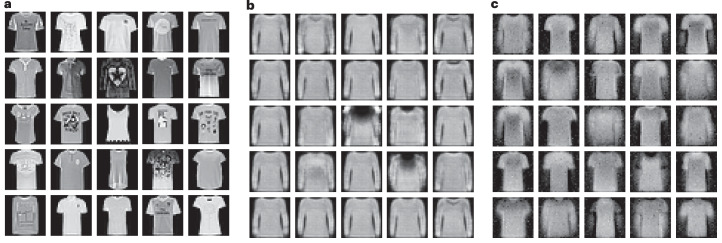
Two-dimensional image generation by a continuous RBM with electrically controlled zero-damping states in isotropic magnets. **a**, Samples of the Fashion-MNIST training data (t-shirt class). **b**,**c**, Generated data using binary and continuous variables for the visible nodes, respectively.

Electrically controlled zero-damping states in isotropic magnets provide a computational resource that samples continuous random variables from the entire Bloch sphere, physically modelling a continuous restricted Boltzmann machine^62,63^ (RBM). We use this model to generate two-dimensional images via an unsupervised machine learning framework. In this scheme, we define visible and hidden layers connected by trainable weights and apply individual biases on each node (see Supplementary Note 9 for more details). The continuous nodes in the visible layer are represented by the magnetization dynamics of an STT-driven isotropic magnet. We trained the continuous RBM and a traditional binary RBM using the Fashion-MNIST benchmark images (Fig. 5a) and at inference, generated new samples, as shown in Fig. 5b,c. The generative artificial intelligence architecture using the continuous variable outperforms those using binary variables by producing more meaningful and diverse images, which we quantitatively demonstrate using standard metrics as detailed in Supplementary Note 9. This improvement arises because, unlike the traditional RBM which is limited to generating binary image data and necessitates averaging during post processing (rate-coded RBM)^64,65^, the continuous RBM directly generates raw image data, enabling smoother convergence and more diverse image generation. Our findings highlight the potential of these magnetic states for various computing applications, superseding conventional binary systems such as stochastic magnetic tunnel junctions acting as probabilistic bits^66^.

Nearly isotropic MgO∣CoFeB∣W multilayers allow a dynamical stabilization of the magnetic order at the maximum of the free energy simply by applying an electric current, creating a research frontier for nonlinear and strongly out-of-equilibrium magnetic properties. Building on our time-averaged measurements on macroscopic samples that reveal the essential physics of this phenomenon, spatially and temporally resolved experiments can test phenomenological, statistical and ultimately quantum-mechanical models. In this regard, microfocus Brillouin light scattering spectroscopy would be a powerful tool^24^. The dynamical stabilization of magnetic order has not yet played a major role in studies of magnonic and spintronic effects^67–69^. However, the option of creating magnetic devices based on isotropic magnets that operate in inverted and dynamically bistable regimes at relatively low power levels provides radically different functionalities that may be useful for, for example probabilistic computation hardware^70^. A robust dynamically stabilized state in the extended film geometry can also lead to experimental studies of recent theoretically predicted antiparticle-like (or anti-magnonic) properties of spin waves around the reversed magnetization state^61^.

*Note added in proof*: Prior to the acceptance of our manuscript, we became aware of a preprint reporting similar results^71^.

## Methods

### Definition of *β*

We parametrize the efficiency of current-induced STTs in the planar SOT geometry by the fit parameter *β* = ℏ*θ*_SH_/(2*eμ*_0_*M*_s_*wd*_M_*d*_W_), where *ℏ*, *e*, *w*, *d*_M_ and *d*_W_ are the reduced Planck constant, the elementary charge, the device bar width and the thicknesses of CoFeB and W layers, respectively.

### Thin film preparation, post-annealing and device fabrication

Thin films were grown on thermally oxidized Si (Si–O/Si) substrates by magnetron sputtering in an ultrahigh vacuum apparatus with the base pressure below 2 × 10^−7^ Pa. The stacking of the thin films was Si substrate∣Si–O∣W (3 nm)∣CoFeB (2 nm)∣MgO (3 nm). All layers were deposited at ambient temperature under the Ar gas pressure of ~5 mTorr. The W and CoFeB (atomic composition of Co_20_Fe_60_B_20_) targets were sputtered using dc power supplies, whereas the MgO target was sputtered using a radio frequency power supply. The deposition rates were set to 0.029 nm s^−1^ for W, 0.022 nm s^−1^ for CoFeB and 0.0016 nm s^−1^ for MgO. The structural characterization of one of the post-annealed W∣CoFeB∣MgO samples was carried out by scanning transmission electron microscopy and shown in Supplementary Note 2. After each sputter-deposition, the films were transferred to the vacuum furnace with the base pressure below 1 × 10^−4^ Pa in which the films were annealed at a target temperature for 1 h—see Supplementary Note 1 for the annealing condition. The thin films were patterned into a rectangle with 10 μm width and 40 μm length by Ar ion milling using standard optical lithography, onto which Au electrodes patterned into a coplanar waveguide were made by sputtering and lift-off techniques.

### ST-FMR and MR measurements

The devices were measured in a microwave prober system to minimize the losses between a microwave signal generator and the device. Typically, we inject microwaves with a power of 13 dBm by the signal generator, with amplitudes modulated by 25% at a frequency of 10 kHz. For ST-FMR measurements, this *I*_mw_sin(2π*ft*) induces a rectification voltage across the device that is measured through a bias tee using a lock-in amplifier set to 10 kHz. *I*_dc_ is applied through the bias tee and a 10 kΩ resistor. The measured data of *V* are fitted using the following functions:

$$V={V}_{0}+{V}_{\mathrm{sym}}\frac{\Delta {H}^{2}}{{({H}_{\mathrm{ext}}-{H}_{\mathrm{res}})}^{2}+\Delta {H}^{2}}+{V}_{\mathrm{asy}}\frac{\Delta H({H}_{\mathrm{ext}}-{H}_{\mathrm{res}})}{{({H}_{\mathrm{ext}}-{H}_{\mathrm{res}})}^{2}+\Delta {H}^{2}},$$

where *V*_0_, *V*_sym_ and *V*_asy_ are the field-independent voltage, the amplitude of the symmetric and antisymmetric components around *H*_res_. We extract *H*_res_ for Fig. 4c by using *H*_ext_ that maximized and minimized the peak amplitude. We measured MR by applying *I*_dc_ through the 10 kΩ resistor in series.

### Macrospin simulations

The LLG equation with a damping-like spin-torque term reads

4 $$\frac{{\rm{d}}{\bf{n}}}{{\rm{d}}t}=-\frac{\gamma }{1+{\alpha }^{2}}{\bf{n}}\times {{\bf{B}}}^{\mathrm{eff}}-\frac{\gamma \alpha }{1+{\alpha }^{2}}{\bf{n}}\times \left({\bf{n}}\times {{\bf{B}}}^{\mathrm{eff}}\right)-\gamma \beta {I}_{\mathrm{dc}}{\bf{n}}\times \left({\bf{n}}\times {\boldsymbol{\sigma }}\right),$$

where **n** is the dimensionless unit vector of the magnetization, **B**^eff^ is the effective field in tesla, *α* = 0.09 is the dimensionless Gilbert damping, *γ* = 1.76 × 10^11^ rad *s*^−1^ T^−1^ is the gyromagnetic ratio and **σ** is the direction of the electron spin polarization. The STT has a strength *β j* with *β* = ℏ*θ*_SH_/(2e*μ*_0_*M*_s_*wd*_M_*d*_W_) = 2.6 × 10^6^ where *θ*_SH_ = 0.22 is the spin-Hall angle, *M*_s_ = 758 kA m^−1^ is the saturation magnetization of CoFeB, *w* = 10 μm is the width of the sample (perpendicular to the current direction) and *d*_W_ = 3 nm is the thickness of the normal metal, and we assume all of the electronic current flows through this layer due to the higher conductivity. *d*_M_ = 1 nm is the thickness of the magnetic layer. We integrate the equation of motion numerically using a fourth-order Runge-Kutta scheme with a time-step Δ*t* = 0.01 ps. The effective field contains terms for the applied field, the demagnetizing effect of a thin film and a PMA that is

5 $${{\bf{B}}}^{\mathrm{eff}}={\mu }_{0}{{\bf{H}}}_{\mathrm{ext}}-\left({\mu }_{0}{M}_{{\rm{s}}}-\frac{2{K}_{\perp }}{{M}_{{\rm{s}}}}\right){{\bf{e}}}_{z}+{\boldsymbol{\xi }},$$

where *μ*_0_ is the vacuum permeability, *μ*_0_**H**_ext_ is the externally applied magnetic field in tesla, *K*_⊥_ = 327 kJ m^−3^ is the magneto-crystalline anisotropy energy density and **e**_*z*_ is a unit vector along the *z*-direction. **ξ** is a stochastic thermal field in tesla with white noise characteristics governed by the fluctuation–dissipation theorem $$\langle {\boldsymbol{\xi }}\rangle =0;\langle \xi (t)\xi (t{{\prime} })\rangle$$$$=\delta (t-t{{\prime} })(2\alpha {k}_{{\rm{B}}}T)$$$$/(\gamma (1+{\alpha }^{2}){M}_{{\rm{s}}}{V}_{{\rm{a}}})$$, where *V*_a_ = 1 × 25 × 25 nm^3^ is the effective volume of a macrospin region in the extended film. We include the effect of Joule heating by renormalizing the anisotropy and magnetization at a given temperature. See Supplementary Note 8 for more details.

## Online content

Any methods, additional references, Nature Portfolio reporting summaries, source data, extended data, supplementary information, acknowledgements, peer review information; details of author contributions and competing interests; and statements of data and code availability are available at 10.1038/s41563-026-02510-z.

## Supplementary information

Supplementary Information Supplementary Notes 1–9 and Figs. 10–14.

## Data Availability

The data presented in the Article and its Supplementary Information are available from the corresponding authors upon reasonable request.

## Code availability

The code utilized in the Article and its Supplementary Information are available from the corresponding authors upon reasonable request.
