# Supplementary material for: Dynamical stability by spin transfer in nearly isotropic magnets
Source: Nat Mater. 2026 Mar 4;25(7):1167–74. doi: 10.1038/s41563-026-02510-z (PMC13323080; doi:10.1038/s41563-026-02510-z)
Supplement: Supplementary file 1 — Supplementary Notes 1–9 and Figs. 10–14. [file 41563_2026_2510_MOESM1_ESM.pdf]

nature materials

Supplementary information

https://doi.org/10.1038/s41563-026-02510-z

Dynamical stability by spin transfer in nearly isotropic magnets

In the format provided by the authors and unedited

CONTENTS

Supplementary Note 1: Minimising the Magnetic Anisotropy
Supplementary Note 2: Scanning transmission electron microscopy characterisation
Supplementary Note 3: magnetoresistance (MR)
Supplementary Note 4: Theoretical Modelling of ST-FMR under Dynamical Stabilisation
Supplementary Note 5: Stability Diagram
Supplementary Note 6: Fokker-Planck Equation
Supplementary Note 7: First Passage Time
Supplementary Note 8: Joule Heating
Supplementary Note 9: Continuous restricted Boltzmann machines using nearly isotropic magnets
Further Supplementary Figures 10-14

Supplementary Note 1: Minimising the Magnetic Anisotropy

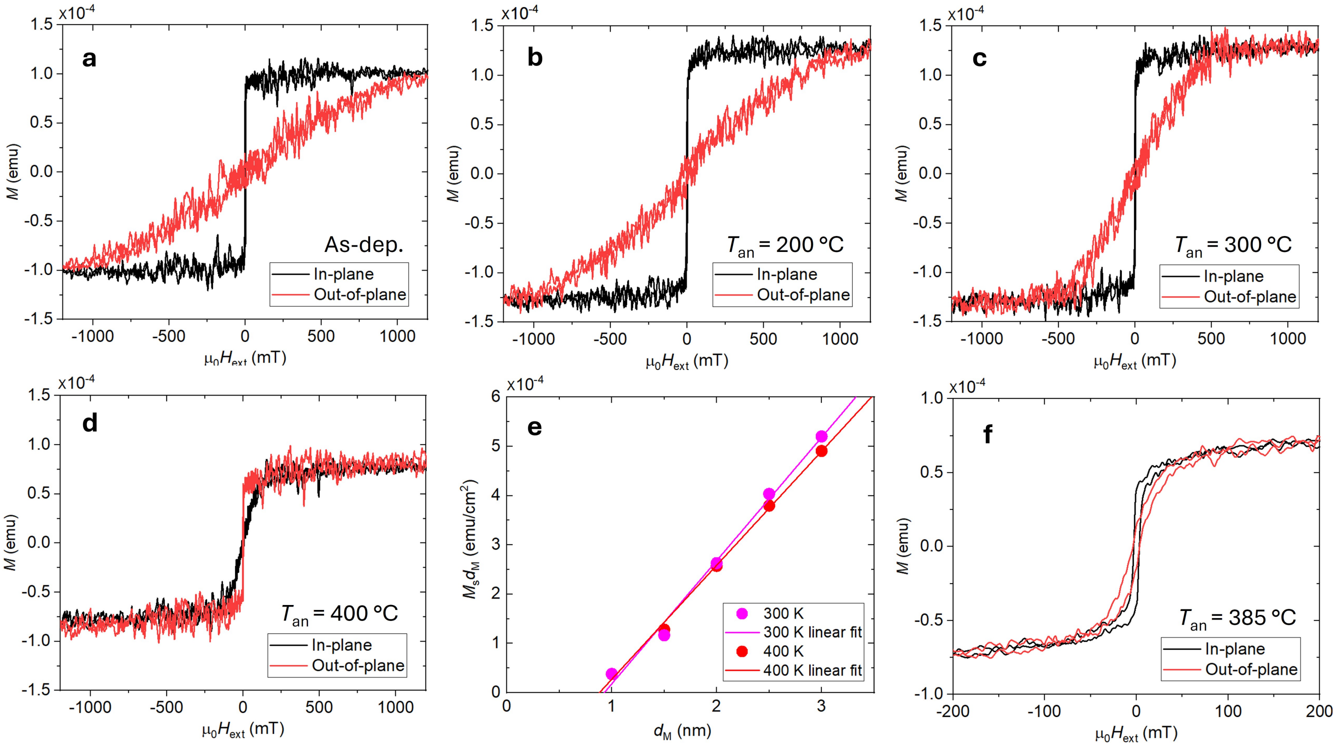

Supplementary Fig. 1. a-d, VSM characterisation of both in-plane and out-of-plane magnetisation-field (M-H) loops of a W|CoFeB (2 nm)|MgO (3 nm) stack for as-deposited (**a**) and subsequently annealed at different temperatures (T_{an}) of 200 °C (**b**), 300 °C (**c**) and 400 °C (**d**). **e**, The product of saturation magnetisation (M_s) extracted by VSM and nominal thickness of CoFeB thickness (d_M) as a function of d_M for the samples after annealed at 300 and 400 °C. **f**, M-H loops for another W|CoFeB (2 nm|MgO (3 nm) sample after annealing at 385 °C.

The magnetic dipole interaction generates an easy-plane magnetic anisotropy in thin films that is proportional to saturation magnetisation (M_s). It competes with the perpendicular anisotropy energy ($K_\perp$) originating from the spin-orbit interaction at the interface between CoFeB and MgO[1,2]. $K_\perp$ can be tuned by various material parameters (e.g. crystallisation, chemical composition and morphology) we can control by post-annealing that only moderately affects M_s. Due to this difference in annealing dependence between $K_\perp$ and M_s, we are able to find an annealing temperature where the effective magnetisation $M_{eff} = M_s - 2K_\perp/(\mu_0 M_s) \approx 0$ (μ_0 is the magnetic permeability of the vacuum). We found that the annealing temperature to realise this condition slightly varies for different grown stacks for our study.

Figures 1a-d show room-temperature M-H loops of a W|CoFeB (2 nm)|MgO stack for different annealing temperatures (T_{an}). We performed this annealing in a vacuum furnace for one hour at T_{an}. We observe a transition from an easy-plane magnet to an out-of-plane easy-axis magnet when increasing T_{an} between 300 °C and 400 °C. We fine-tuned the annealing temperature to minimise M_{eff} for CoFeB thicknesses (d_M) and plot the product of M_s and d_M as a function of d_M in Fig. 1e. These results point to a magnetically dead layer of approximately 1 nm. Figure 1f displays M-H loops for another W|CoFeB (2 nm)|MgO (3 nm) sample for T_{an} = 385 °C before we patterned this stack into transport devices that provide results for the main manuscript.

Supplementary Note 2: Scanning transmission electron microscopy characterisation

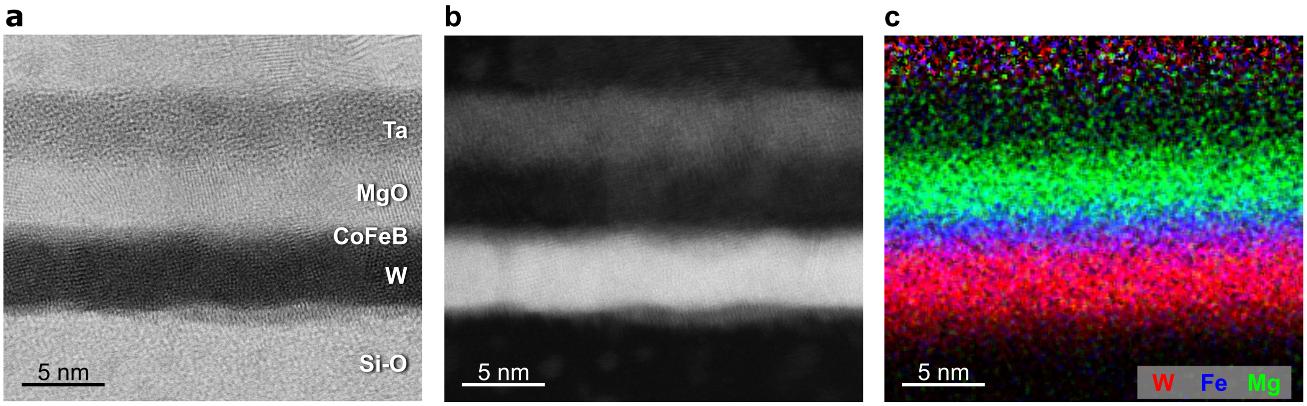

Supplementary Fig. 2. a-b, Cross-sectional BF (**a**) and HAADF (**b**) STEM images of the W|CoFeB|MgO stack. **c**, STEM-EDS elemental maps of the sample.

The structural characterisation of the post-annealed W|CoFeB|MgO sample was carried out using scanning transmission electron microscopy (STEM). A cross-sectional STEM specimen was prepared from an Si substrate|Si-O|W|CoFeB|MgO stacks with a Ta capping layer using focused ion beam systems. The STEM observation was performed using a JEM-ARM200F (JEOL Ltd.) equipped with an energy dispersive x-ray spectroscopy (EDS) detector. Figures 2a-b show the cross-sectional bright field (BF) and high-angle annular dark-field (HAADF) STEM images, respectively, where we confirm clear contrasts for W and MgO layers owing to their relatively large thicknesses. The CoFeB layer can also be recognised between the two layers where partial crystallisation of CoFeB due to the post-annealing is observed. EDS elemental mapping in Fig. 2c further confirms the presence of Fe atoms, indicating that the clear layer stacking structure of W|CoFeB|MgO has been achieved.

Supplementary Note 3: magnetoresistance (MR)

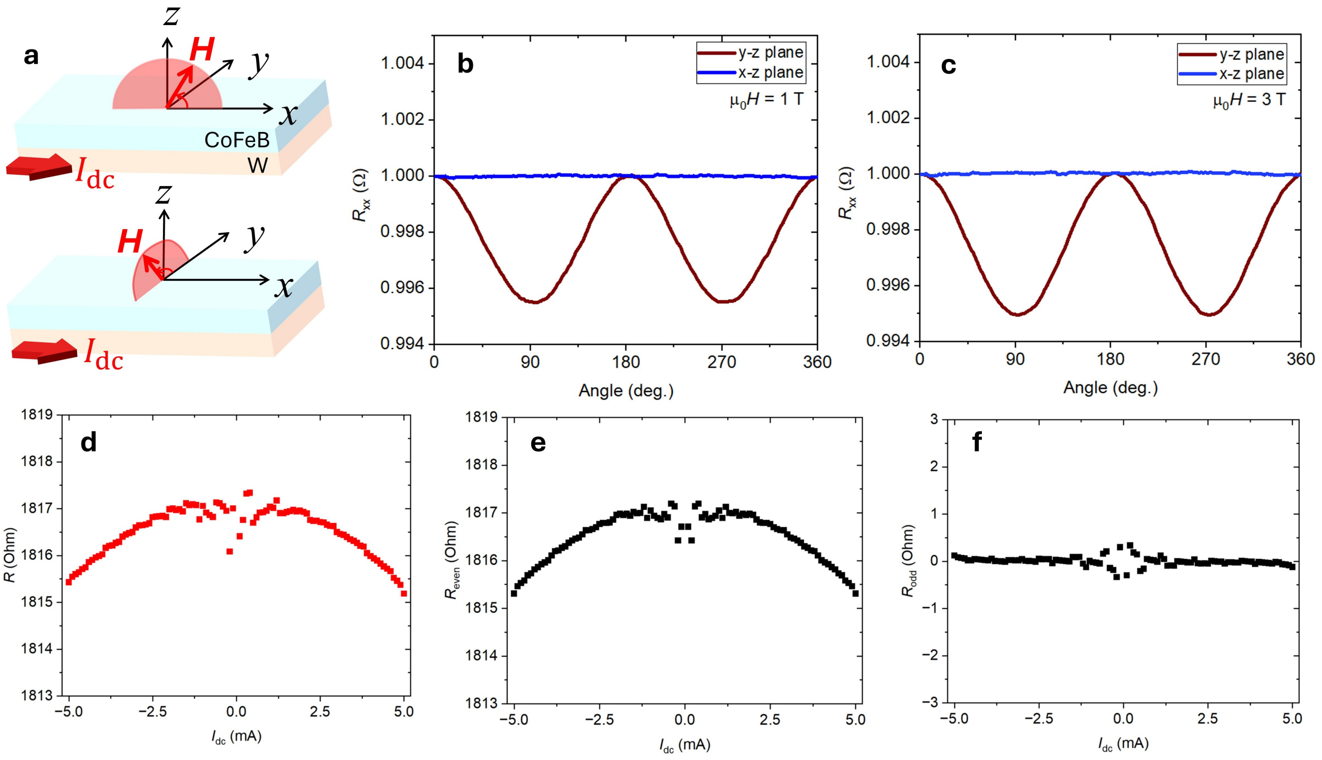

Supplementary Fig. 3. **a**, magnetoresistance configurations for x-z (top) and y-z (bottom) field rotations while a dc electric current (I_{dc}) flows along the x direction for both cases. **b-c**, Fixed-field angular-dependent magnetoresistance measurements at the magnetic fields of 1 T (**b**) and 3 T (**c**) respectively, and performed at room temperature. **d**, Resistance (R) measurements by sweeping dc currents (I_{dc}) while applying a constant magnetic field at 516 mT. Artifact due to a small residual current from the source meter has been extracted before plotting R. **e(f)**, the even(odd) component R_{even}(R_{odd}) of R with respect to I_{dc} reversal. The scale of the resistance change is fixed for 6 Ohms to compare the magnitude of the resistance change directly side by side.

We measured the room temperature MR of MgO(3 nm)|CoFeB(2 nm)|W(3 nm) stacks as shown in Fig. 3. Because both CoFeB and W layers are metallic, the anisotropic magnetoresistance (AMR)[3] in CoFeB coexists with spin-Hall magnetoresistance (SMR)[4,5] in W. Although these mechanisms cannot be distinguished when rotating the magnetisation in the film plane (e.g. those in Fig. 3 of the main manuscript), two high-symmetry out-of-plane rotations (Fig. 3a) do lead to different angular dependencies to quantify individual components experimentally. The AMR depends on the angle between the magnetisation and the current directions (the x direction in the Fig. 3a schematics), whereas the SMR senses the angle between magnetisation and the polarisation of the spin Hall current (in our case along the y direction). In Fig. 3(c), the MR in the y-z plane for a constant field of 1 T(3 T) oscillates, while the MR in the x-z scan is negligibly small, i.e. the SMR dominates. Because of this, we rule out the current-induced self-torque in the CoFeB layer from our analysis.

Next, we discuss the relationship between our measurements presented in Fig. 3 in the main text and the unidirectional magnetoresistance (UMR)[6], or more specifically unidirectional SMR[7]. Among different mechanisms of UMR[6–9], the magnon contribution is responsible for the leading order I_{dc} dependence of $\langle M_y^2 \rangle$. When the magnetisation dynamics has large amplitudes, $\langle M_y^2 \rangle$ as a function of I_{dc} is highly non-linear and contains both odd and even components, see Fig. 3a-b. To assess UMR components that are unrelated to magnons, we measured the resistance at a high magnetic field of 516 mT that strongly suppresses the current-induced modulation of $\langle M_y^2 \rangle$. In Fig. 3d we plot the resistance R (after subtracting an instrumental artifact caused by a finite residual current in the source meter). The UMR contribution to R is odd under current reversal and captured by $R_{odd}(I_{dc}) = (R(I_{dc}) - R(-I_{dc}))/2$. However, Fig. 3e-f shows an I_{dc} dependence of R that is dominated by the even component $R_{even}(I_{dc}) = (R(I_{dc}) + R(-I_{dc}))/2$, which signals Joule heating. Repeating these measurements at 618, 718, 816, 902 and 978 mT (data not shown) confirm that a non-magnonic contribution to the UMR is negligibly small.

Supplementary Note 4: Theoretical Modelling of ST-FMR under Dynamical Stabilisation

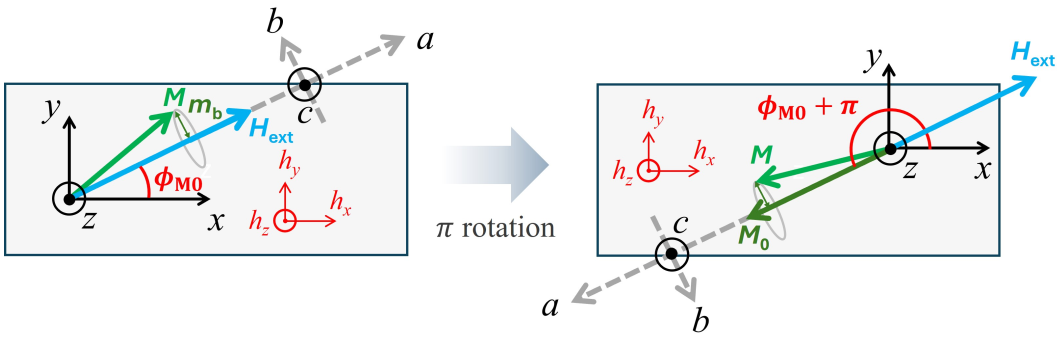

Supplementary Fig. 4. The coordinate systems used in our study are shown and both schematics are top-views of our devices. For our analysis of magnetisation dynamics, we use the *a*-*b*-*c* coordinates defined by the direction of magnetic field, in other words, the direction of the time-averaged moment for the *a* axis. Due to this definition, this coordinate system depends on the orientation of magnetic field and here we show the case of magnetisation reversal where the *b* axis has been flipped.

We start from Landau-Lifshitz-Gilbert equation for the macrospin augumented by the SOT[10]

$$\frac{d\boldsymbol{M}}{dt} = -\gamma\mu_0 \boldsymbol{M} \times \left(\boldsymbol{H}_{\text{ext}} - M_{\text{eff}}\frac{M_z}{M_{\text{s}}}\hat{\boldsymbol{z}} + \boldsymbol{h}\right) + \frac{\alpha}{M_{\text{s}}}\boldsymbol{M} \times \frac{d\boldsymbol{M}}{dt} - \gamma\mu_0\beta\left(I_{\text{dc}} + I_{\text{mw}}\cos\omega t\right)\frac{\boldsymbol{M}}{M_{\text{s}}} \times (\boldsymbol{M} \times \hat{\boldsymbol{y}}), \tag{1}$$

with symbols defined in the main text, except for $\omega = 2\pi f$ and the rf Oersted field $\boldsymbol{h} = h\hat{\boldsymbol{y}}$ generated by the charge current[10]. We disregard here the spin waves with nonzero wavelengths and thermal fluctuations. If the SOT is caused by the injection of the spin current generated by the spin-Hall effect, $\beta = \hbar\theta_{\text{SH}}/2e\mu_0 M_{\text{s}} w d_{\text{M}} d_{\text{W}}$ where $\hbar, \theta_{\text{SH}}, e, w, d_{\text{M}}, d_{\text{W}}$ are the reduced Planck constant, spin-Hall angle of W, elementary electric charge, width of the multilayer stack, and the thicknesses of the CoFeB and W layers respectively. In our setup, the W|CoFeB interface may well play a crucial role in deciding the magnitude of SOT, so that we choose to use the same symbol β to avoid specifying the microscopic mechanism of SOT. We write $\boldsymbol{M} = \boldsymbol{M}_0 + \delta\boldsymbol{m}$ where $\boldsymbol{M}_0$ is independent of time, and treat $\delta\boldsymbol{m}$ as a small perturbation. Denoting the Fourier transform in time by a tilde, the linearized LLG equation reads

$$\left(i\omega - \kappa + \gamma\mu_0\beta I_{\text{dc}}\frac{M_{0y}}{M_{\text{s}}}\right)\widetilde{\delta\boldsymbol{m}} + \left(\gamma\mu_0\boldsymbol{H}_{\text{ext}} - i\alpha\omega\frac{\boldsymbol{M}_0}{M_{\text{s}}}\right) \times \widetilde{\delta\boldsymbol{m}} = \gamma\mu_0\frac{\boldsymbol{M}_0}{M_{\text{s}}} \times \widetilde{\boldsymbol{h}} + \gamma\mu_0\beta\widetilde{I}_{\text{mw}}\frac{\boldsymbol{M}_0}{M_{\text{s}}} \times \left(\frac{\boldsymbol{M}_0}{M_{\text{s}}} \times \hat{\boldsymbol{y}}\right), \tag{2}$$

where $\widetilde{I}_{\text{mw}} = I_{\text{mw}}/2$ and $\kappa > 0$ is a phenomenological relaxation rate on top of the Gilbert damping that may depend on the reference state $\boldsymbol{M}_0$ around which the linearisation is carried out. $\kappa > 0$ acts as an additional stabilisation of the reference state. The atomistic simulations did not include κ, which is therefore not essential to achieve the dynamical stabilization of the anti-parallel state. However, we keep it here because the fitting of experimental data indicates the presence of additional dissipation processes beyond the Gilbert damping. Introducing polar coordinates of the Bloch sphere

$$\frac{\boldsymbol{M}_0}{M_{\text{s}}} = \begin{pmatrix} \sin\theta_M\cos\phi_M \\ \sin\theta_M\sin\phi_M \\ \cos\theta_M \end{pmatrix}, \tag{3}$$

we define a ground-state-adapted Cartesian frame $\{\boldsymbol{a}, \boldsymbol{b}, \boldsymbol{c}\}$ (illustrated in Fig. 4 for $\theta_M = \pi/2$) as

$$\boldsymbol{a} = \frac{\boldsymbol{M}_0}{M_{\text{s}}}, \quad \boldsymbol{b} = \begin{pmatrix} -\sin\phi_M \\ \cos\phi_M \\ 0 \end{pmatrix}, \quad \boldsymbol{c} = \boldsymbol{a} \times \boldsymbol{b} = \begin{pmatrix} -\cos\theta_M\cos\phi_M \\ -\cos\theta_M\sin\phi_M \\ \sin\theta_M \end{pmatrix}. \tag{4}$$

With $H^a_{\text{ext}} = \boldsymbol{H}_{\text{ext}} \cdot \boldsymbol{a}, H^b_{\text{ext}} = \boldsymbol{H}_{\text{ext}} \cdot \boldsymbol{b}$, etc., Eq. (2) becomes

$$\begin{pmatrix} H^a_{\text{ext}} - M_{\text{eff}}\cos^2\theta_M - \frac{i\alpha\omega}{\gamma\mu_0} & \frac{i\omega-\kappa}{\gamma\mu_0} - \beta I_{\text{dc}}\sin\theta_M\sin\phi_M \\ \frac{-i\omega+\kappa}{\gamma\mu_0} + \beta I_{\text{dc}}\sin\theta_M\sin\phi_M & H^a_{\text{ext}} - M_{\text{eff}}\cos 2\theta_M - \frac{i\alpha\omega}{\gamma\mu_0} \end{pmatrix}\begin{pmatrix} \widetilde{\delta m^b} \\ \widetilde{\delta m^c} \end{pmatrix} = \begin{pmatrix} \widetilde{h}\cos\phi_M + \beta\widetilde{I_{\text{mw}}}\cos\theta_M\sin\phi_M \\ -\widetilde{h}\cos\theta_M\sin\phi_M + \beta\widetilde{I_{\text{mw}}}\cos\phi_M \end{pmatrix}. \tag{5}$$

To the leading order terms in α, κ/ω, and β

$$\widetilde{\delta m^b} = \frac{\left\{\left(H^a_{\rm ext}-M_{\rm eff}\cos 2\theta_M\right)\cos\phi_M+\frac{i\omega}{\gamma\mu_0}\cos\theta_M\sin\phi_M\right\}\tilde{h}+\left\{\left(H^a_{\rm ext}-M_{\rm eff}\cos 2\theta_M\right)\cos\theta_M\sin\phi_M-\frac{i\omega}{\gamma\mu_0}\cos\phi_M\right\}\beta\widetilde{I_{\rm mw}}}{\left(H^a_{\rm ext}-H^+_{\rm res}\right)\left(H^a_{\rm ext}+H^-_{\rm res}\right)-\frac{i\omega}{\gamma\mu_0}\left\{\alpha\left(2H^a_{\rm ext}-H^+_{\rm res}+H^-_{\rm res}\right)+\frac{2\kappa}{\gamma\mu_0}+\beta I_{\rm dc}\sin\theta_M\sin\phi_M\right\}}, \tag{6}$$

$$\widetilde{\delta m^c} = \frac{-\left\{\left(H^a_{\rm ext}-M_{\rm eff}\cos^2\theta_M\right)\cos\theta_M\sin\phi_M-\frac{i\omega}{\gamma\mu_0}\cos\phi_M\right\}\tilde{h}+\left\{\left(H^a_{\rm ext}-M_{\rm eff}\cos^2\theta_M\right)\cos\phi_M+\frac{i\omega}{\gamma\mu_0}\cos\theta_M\sin\phi_M\right\}\beta\widetilde{I_{\rm mw}}}{\left(H^a_{\rm ext}-H^+_{\rm res}\right)\left(H^a_{\rm ext}+H^-_{\rm res}\right)-\frac{i\omega}{\gamma\mu_0}\left\{\alpha\left(2H^a_{\rm ext}-H^+_{\rm res}+H^-_{\rm res}\right)+\frac{2\kappa}{\gamma\mu_0}+\beta I_{\rm dc}\sin\theta_M\sin\phi_M\right\}}, \tag{7}$$

where we introduced the provisional resonance fields $H^{\pm}_{\rm res}$ for the thermal equilibrium (+) and dynamically stabilised (-) state

$$H^{\pm}_{\rm res} = \sqrt{\frac{\omega^2}{\gamma^2\mu_0^2}+\frac{M^2_{\rm eff}\sin^4\theta_M}{4}} \pm \frac{M_{\rm eff}\left(3\cos^2\theta_M-1\right)}{2}. \tag{8}$$

The SMR under applied currents $I_{\rm dc}, I_{\rm mw}\cos\omega t$ generates a dc voltage

$$V_{\rm dc} = \left\{R_0+\Delta R_{\rm SMR}\left(1-\sin^2\theta_M\sin^2\phi_M\right)\right\}I_{\rm dc} - \frac{2\Delta R_{\rm SMR}}{M_{\rm s}}I_{\rm mw}\,{\rm Re}\left[\widetilde{\delta m_y}\right]\sin\theta_M\sin\phi_M. \tag{9}$$

With $\delta m_y = \delta m^b\cos\phi_M - \delta m^c\cos\theta_M\sin\phi_M$,

$$\begin{aligned}{\rm Re}\left[\widetilde{\delta m_y}\right] =& {\rm Re}\left[\frac{\left(H^a_{\rm ext}-M_{\rm eff}\cos 2\theta_M\right)\cos^2\phi_M+\left(H^a_{\rm ext}-M_{\rm eff}\cos^2\theta_M\right)\cos^2\theta_M\sin^2\phi_M}{\left(H^a_{\rm ext}-H^+_{\rm res}\right)\left(H^a_{\rm ext}+H^-_{\rm res}\right)-\frac{i\omega}{\gamma\mu_0}\left\{\alpha\left(2H^a_{\rm ext}-H^+_{\rm res}+H^-_{\rm res}\right)+\frac{2\kappa}{\gamma\mu_0}+\beta I_{\rm dc}\sin\theta_M\sin\phi_M\right\}}\right]\tilde{h} \\ &+{\rm Im}\left[\frac{\frac{\omega}{\gamma\mu_0}\left(\cos^2\phi_M+\cos^2\theta_M\sin^2\phi_M\right)+iM_{\rm eff}\cos\theta_M\sin^2\theta_M\cos\phi_M\sin\phi_M}{\left(H^a_{\rm ext}-H^+_{\rm res}\right)\left(H^a_{\rm ext}+H^-_{\rm res}\right)-\frac{i\omega}{\gamma\mu_0}\left\{\alpha\left(2H^a_{\rm ext}-H^+_{\rm res}+H^-_{\rm res}\right)+\frac{2\kappa}{\gamma\mu_0}+\beta I_{\rm dc}\sin\theta_M\sin\phi_M\right\}}\right]\beta\widetilde{I_{\rm mw}},\end{aligned} \tag{10}$$

where $\tilde{h}$ is real since h is in-phase with $I_{\rm mw}\cos\omega t$. This formula holds for arbitrary direction of $\boldsymbol{M}_0$, which does not necessarily behave like a simple Lorentzian.

Next, we focus on $M_{\rm eff} > 0$ and $\boldsymbol{M}_0 \parallel \pm\boldsymbol{H}_{\rm ext}$ *i.e.*, $\theta_M = \pi/2$ and $\phi_M = \phi$ or $\phi_M = \phi + \pi$. The stability of these reference states will be studied in the next section. $\boldsymbol{M}_0 \parallel \pm\boldsymbol{H}_{\rm ext}$ implies $H^a_{\rm ext} = \pm H_{\rm ext}$, and different resonance fields $H^{\pm}_{\rm res}$ for the parallel and anti-parallel states respectively. Assuming constant θ_M, ϕ_M, one can replace $H^a_{\rm ext}$ in Eq. (10) by $\pm H^{\pm}_{\rm res}$ except in the terms rapidly approaching zero in the denominators close to the resonance. We recover the Lorentzian dependence on $H_{\rm ext}$ when the imaginary part of the denominator is constant. However, we tune $I_{\rm dc}$ such that the imaginary part vanishes at a field near the resonance. The experiment indeed shows deviations from the Lorentzian lineshape, which is qualitatively consistent with Eq. (10). In the following, we disregard this complication for simplicity by using the expression $V_{\rm dc} = \left\{R_0+\Delta R_{\rm SMR}\left(1-\sin^2\phi\right)\right\}I_{\rm dc}+V_{\rm sym}+V_{\rm asy}$ with $V_{\rm sym}, V_{\rm asy}$ the symmetric and anti-symmetric parts of the ST-FMR signal given by

$$V_{\rm sym} = -2\Delta R_{\rm SMR}\beta\widetilde{I_{\rm mw}}^2\sqrt{\frac{1}{1+\gamma^2\mu_0^2M^2_{\rm eff}/4\omega^2}}\frac{\pm\Delta H_{\pm}\cos^2\phi\sin\phi}{(H_{\rm ext}-H^{\pm}_{\rm res})^2+\Delta H^2_{\pm}}, \tag{11}$$

$$V_{\rm asy} = -4\Delta R_{\rm SMR}\widetilde{I_{\rm mw}}\tilde{h}\frac{H^{\pm}_{\rm res}\pm M_{\rm eff}}{H^+_{\rm res}+H^-_{\rm res}}\frac{\left(H_{\rm ext}-H^{\pm}_{\rm res}\right)\cos^2\phi\sin\phi}{(H_{\rm ext}-H^{\pm}_{\rm res})^2+\Delta H^2_{\pm}}, \tag{12}$$

where the linewidths for the parallel and anti-parallel reference states read

$$\Delta H_{\pm} = \Delta H_0 \pm \frac{\alpha\omega}{\gamma\mu_0} \pm \sqrt{\frac{1}{1+\gamma^2\mu_0^2M^2_{\rm eff}/4\omega^2}}\beta I_{\rm dc}\sin\phi, \quad \Delta H_0 = \frac{\kappa}{\gamma\mu_0}\sqrt{\frac{1}{1+\gamma^2\mu_0^2M^2_{\rm eff}/4\omega^2}}. \tag{13}$$

Although we used the notation ΔH_0 to suggest it corresponds to the inhomogeneous broadening, κ was not introduced as such and we regard both ΔH_0 and κ as pure fitting parameters without any association to underlying physics. The FMR experiments are well represented by a symmetric Lorentzian (*i.e.*, $V_{\rm sym} \gg V_{\rm asy}$), which indicates that field-like torques are very small *i.e.*, $\beta|I_{\rm mw}| \gg |\boldsymbol{h}|$. For fixed ϕ, the angle for the magnetic field direction, we obtain a familiar expression of linewidth in the parallel state ΔH_+ in which $I_{\rm dc}\sin\phi < 0$ counteracts the Gilbert damping. The linewidth of the anti-parallel state ΔH_- is negative when SOT and ΔH_0 vanish, reflecting that it is unstable at a maximum of the free energy. A large negative $I_{\rm dc}\sin\phi$ turns ΔH_- positive, which indicates stability of the anti-parallel state that can be detected by a sign change of $V_{\rm sym}$. In the next section, we will show that for a sufficiently large negative $I_{\rm dc}\sin\phi$, $\Delta H_- > 0$ for $H_{\rm ext}$ near $H^-_{\rm res}$ so that the sign of $V_{\rm sym}$ indicates whether $\boldsymbol{M}_0$ is parallel or anti-parallel to $\boldsymbol{H}_{\rm ext}$. Substituting $\omega = 2\pi f$ into Eqs. (11) and (13) yields Eqs. (2) and (3) in the main text respectively.

Supplementary Note 5: Stability Diagram

In this section, we describe how the bifurcation diagram Fig. 4a in the main text is generated. Namely, we analytically determine the conditions for stability of various time-independent solutions of Eq. (1), referred to as fixed points, for $\boldsymbol{H}_{\text{ext}} = H_{\text{ext}}\hat{\boldsymbol{y}}$ and $h = I_{\text{mw}} = 0$. For $\phi \neq \pm\pi/2$, the stability conditions should be well approximated by replacing I_{dc} in the formulae for $\phi = \pm\pi/2$ by $I_{\text{dc}} \sin\phi$ with the deviation proportional to $\beta I_{\text{dc}}/(H_{\text{ext}} \pm M_{\text{eff}})$ that we assume to be small. We first enumerate all the possible fixed points in the polar coordinates Eq. (3) by setting $d\boldsymbol{M}/dt = 0$ in Eq. (1), which leads to

$$\begin{pmatrix} 1 & \eta_{\text{STT}}\cos\theta_M \\ -\eta_{\text{STT}} & \cos\theta_M \end{pmatrix} \begin{pmatrix} \cos\phi_M \\ \sin\phi_M \end{pmatrix} = -\nu \begin{pmatrix} 0 \\ \cos\theta_M \sin\theta_M \end{pmatrix}, \tag{14}$$

where $\eta_{\text{STT}} = \beta I_{\text{dc}}/H_{\text{ext}}$ is the dimensionless STT efficiency and $\nu = M_{\text{eff}}/H_{\text{ext}}$ the anisotropy. The solutions with $\cos\theta_M = 0$, *i.e.*, magnetisation in the plane, are the parallel and anti-parallel states $\phi_M = \pm\pi/2$. For $\cos\theta_M \neq 0$, the canting angle θ_M solves the equation

$$\eta_{\text{STT}}^2 \sin^4\theta_M - \left(1 + \eta_{\text{STT}}^2\right)\sin^2\theta_M + \frac{1}{\nu^2}\left(1 + \eta_{\text{STT}}^2\right)^2 = 0. \tag{15}$$

We restrict ourselves to relatively small currents with $-1 < \eta_{\text{STT}} < 1$. A solution, which is unique up to the sign, exists when $|\nu| > 1 + \eta_{\text{STT}}^2$;

$$\sin^2\theta_M = \frac{1 + \eta_{\text{STT}}^2}{2\eta_{\text{STT}}^2}\left(1 - \sqrt{1 - \frac{4\eta_{\text{STT}}^2}{\nu^2}}\right), \quad \begin{pmatrix} \cos\phi_M \\ \sin\phi_M \end{pmatrix} = -\frac{\nu\sin\theta_M}{1 + \eta_{\text{STT}}^2}\begin{pmatrix} -\eta_{\text{STT}}\cos\theta_M \\ 1 \end{pmatrix}. \tag{16}$$

For $M_{\text{eff}} < 0$, we recover the solution at thermal equilibrium in which the in-plane H_{ext} and the PMA compete. To summarise, the fixed points are: (i) the thermal equilibrium state $\theta_M = \pi/2, \phi_M = \pi/2$, (ii) the inverted state $\theta_M = \pi/2, \phi_M = -\pi/2$, and (iii) the pair of out-of-plane states given in Eq. (16) that exist only for a sufficiently large $|M_{\text{eff}}|$. Thier stability follows from Eq. (5) with right-hand-sides set to zero, which leads to complex eigenfrequencies

$$\begin{aligned} \frac{(1+\alpha^2)\,\omega}{\gamma\mu_0 H_{\text{ext}}} = & -i\left\{\alpha\left(\sin\theta_M\sin\phi_M - \nu\frac{3\cos^2\theta_M - 1}{2}\right) + \frac{\kappa}{\gamma\mu_0 H_{\text{ext}}} + \eta_{\text{STT}}\sin\theta_M\sin\phi_M\right\} \\ & \pm\left[\left\{\sin\theta_M\sin\phi_M - \nu\cos^2\theta_M - \alpha\left(\frac{\kappa}{\gamma\mu_0 H_{\text{ext}}} + \eta_{\text{STT}}\sin\theta_M\sin\phi_M\right)\right\}\right. \\ & \left.\times\left\{\sin\theta_M\sin\phi_M - \nu\cos 2\theta_M - \alpha\left(\frac{\kappa}{\gamma\mu_0 H_{\text{ext}}} + \eta_{\text{STT}}\sin\theta_M\sin\phi_M\right)\right\} - \frac{\alpha^2\nu^2\sin^4\theta_M}{4}\right]^{1/2}. \end{aligned} \tag{17}$$

The fixed point is stable if and only if the imaginary parts of both frequencies are negative.

1. Parallel state

The eigenfrequencies take the familiar form

$$\begin{aligned} \frac{(1+\alpha^2)\,\omega}{\gamma\mu_0 H_{\text{ext}}} = & -i\left\{\alpha\left(1 + \frac{\nu}{2}\right) + \frac{\kappa}{\gamma\mu_0 H_{\text{ext}}} + \eta_{\text{STT}}\right\} \\ & \pm\sqrt{\left\{1 - \alpha\left(\frac{\kappa}{\gamma\mu_0 H_{\text{ext}}} + \eta_{\text{STT}}\right)\right\}\left\{1 + \nu - \alpha\left(\frac{\kappa}{\gamma\mu_0 H_{\text{ext}}} + \eta_{\text{STT}}\right)\right\} - \frac{\alpha^2\nu^2}{4}}. \end{aligned} \tag{18}$$

The fixed point is stable if (i) the imaginary part of the first term on the right-hand-side is negative

$$-\eta_{\text{STT}} < \alpha\left(1 + \frac{\nu}{2}\right) + \frac{\kappa}{\gamma\mu_0 H_{\text{ext}}}, \tag{19}$$

and (ii) the modulus of the imaginary part of the square root is smaller than that of the first term, *i.e.*,

$$-\left(1 + \alpha^2\right)\left\{1 + \nu + \left(\frac{\kappa}{\gamma\mu_0 H_{\text{ext}}} + \eta_{\text{STT}}\right)^2\right\} < 0. \tag{20}$$

Both conditions are satisfied for an easy-plane anisotropy $M_{\text{eff}} > 0$ and a sufficiently small STT. The parallel state can be destabilised by a large negative STT or by an easy-axis anisotropy $M_{\text{eff}} \lesssim -H_{\text{ext}}$. Equation (1) in the main text is derived by replacing "<" by "=" in Eq. (19) and multiplying both sides by H_{ext}, under the identification $\Delta H_0' = \kappa/(\gamma\mu_0)$.

2. Anti-parallel state

Substituting $\theta_M = \pi/2, \phi_M = -\pi/2$ into Eq. (17) yields

$$\begin{aligned}\frac{(1+\alpha^2)\,\omega}{\gamma\mu_0 H_{\text{ext}}} &= i\left\{\alpha\left(1-\frac{\nu}{2}\right)-\frac{\kappa}{\gamma\mu_0 H_{\text{ext}}}+\eta_{\text{STT}}\right\}\\ &\quad \pm\sqrt{\left\{1+\alpha\left(\frac{\kappa}{\gamma\mu_0 H_{\text{ext}}}-\eta_{\text{STT}}\right)\right\}\left\{1-\nu+\alpha\left(\frac{\kappa}{\gamma\mu_0 H_{\text{ext}}}-\eta_{\text{STT}}\right)\right\}-\frac{\alpha^2\nu^2}{4}}.\end{aligned} \tag{21}$$

As argued above, the stability conditions are

$$-\eta_{\text{STT}} > \alpha\left(1-\frac{\nu}{2}\right)-\frac{\kappa}{\gamma\mu_0 H_{\text{ext}}}, \tag{22}$$

and

$$-\left(1+\alpha^2\right)\left\{1-\nu+\left(\frac{\kappa}{\gamma\mu_0 H_{\text{ext}}}-\eta_{\text{STT}}\right)^2\right\} < 0. \tag{23}$$

A large κ may by itself stabilise the state, which is simply how the phenomenological relaxation was introduced. We do not know whether this is a sensible prescription, but keep this parameter for the sake of uniformity across different fixed points. In practice, it becomes important only for fitting the experimental data with the results concerning the parallel fixed point. When $\alpha H_{\text{ext}} > \kappa/\gamma\mu_0$, the condition (22) can be satisfied either for large negative η_{STT} or large positive M_{eff}. Since the latter violates the second condition (23), $\eta_{\text{STT}} < 0$ is a necessary condition for the stability.

3. Out-of-plane states

When using Eq. (16) in Eq. (17)

$$\begin{aligned}\frac{(1+\alpha^2)\,\omega}{\gamma\mu_0 H_{\text{ext}}} &= i\nu\left\{\alpha-\frac{\alpha-2\eta_{\text{STT}}+3\alpha\eta_{\text{STT}}^2}{4\eta_{\text{STT}}^2}\left(1-\sqrt{1-\frac{4\eta_{\text{STT}}^2}{\nu^2}}\right)-\frac{\kappa}{\gamma\mu_0 M_{\text{eff}}}\right\}\\ &\quad \pm\nu\left[\left\{1-\frac{\eta_{\text{STT}}(\alpha+\eta_{\text{STT}})}{2\eta_{\text{STT}}^2}\left(1-\sqrt{1-\frac{4\eta_{\text{STT}}^2}{\nu^2}}\right)+\frac{\alpha\kappa}{\gamma\mu_0 M_{\text{eff}}}\right\}\right.\\ &\quad \left.\times\left\{1-\frac{1+\eta_{\text{STT}}(\alpha+2\eta_{\text{STT}})}{2\eta_{\text{STT}}^2}\left(1-\sqrt{1-\frac{4\eta_{\text{STT}}^2}{\nu^2}}\right)+\frac{\alpha\kappa}{\gamma\mu_0 M_{\text{eff}}}\right\}-\frac{\alpha^2\sin^4\theta_M}{4}\right]^{1/2}.\end{aligned} \tag{24}$$

The conditions for stability are (i) the imaginary part of the first term to be negative

$$\nu\left\{\alpha-\frac{\alpha-2\eta_{\text{STT}}+3\alpha\eta_{\text{STT}}^2}{4\eta_{\text{STT}}^2}\left(1-\sqrt{1-\frac{4\eta_{\text{STT}}^2}{\nu^2}}\right)\right\} < \frac{\kappa}{\gamma\mu_0 H_{\text{ext}}} \tag{25}$$

and (ii) the modulus of the imaginary part of the square root to be smaller than that of the first line

$$\left\{1+\frac{1}{2}\left(1-\sqrt{1-\frac{4\eta_{\text{STT}}^2}{\nu^2}}\right)\right\}\left\{1-\frac{1-\eta_{\text{STT}}^2}{2\eta_{\text{STT}}^2}\left(1-\sqrt{1-\frac{4\eta_{\text{STT}}^2}{\nu^2}}\right)\right\}+\left(\frac{\kappa}{\gamma\mu_0 M_{\text{eff}}}-\frac{1}{2\eta_{\text{STT}}}\left(1-\sqrt{1-\frac{4\eta_{\text{STT}}^2}{\nu^2}}\right)\right)^2 > 0. \tag{26}$$

The latter condition is satisfied whenever the fixed points exist since

$$\frac{1-\eta_{\text{STT}}^2}{2\eta_{\text{STT}}^2}\left(1-\sqrt{1-\frac{4\eta_{\text{STT}}^2}{\nu^2}}\right) < \sin^2\theta_M \leq 1. \tag{27}$$

When $\eta_{\text{STT}} = 0$, those fixed points are stable equilibria only for an easy-axis anisotropy $M_{\text{eff}} < 0$. For $\eta_{\text{STT}}^2 < 1$ and $\alpha \ll 1$, the STT reinforces or counteracts the Gilbert damping according to whether $\eta_{\text{STT}} > 0$ or $\eta_{\text{STT}} < 0$. Therefore and counterintuitively, a large negative η_{STT} can stabilise out-of-plane states even for $M_{\text{eff}} > 0$ or destabilise them when $M_{\text{eff}} < 0$.

4. Bifurcation diagram

The results of the previous subsections can be summarised in the space spanned by the dimensionless parameters η_{STT} and ν. The separatrices, *i.e.*, the curves across which the stability of at least one of the fixed points changes, read (upper inequality for stability):

Parallel state

$$\nu \gtrless -2 - \frac{2\eta_{\mathrm{STT}}}{\alpha} - \frac{2\kappa}{\gamma\mu_0 H_{\mathrm{ext}}}, \tag{28}$$

$$\nu \gtrless -1 - \left(\eta_{\mathrm{STT}} + \frac{\kappa}{\gamma\mu_0 H_{\mathrm{ext}}}\right)^2. \tag{29}$$

Anti-parallel state

$$\nu \gtrless 2 + \frac{2\eta_{\mathrm{STT}}}{\alpha} - \frac{2\kappa}{\gamma\mu_0 H_{\mathrm{ext}}}, \tag{30}$$

$$\nu \lessgtr 1 + \left(\eta_{\mathrm{STT}} - \frac{\kappa}{\gamma\mu_0 H_{\mathrm{ext}}}\right)^2. \tag{31}$$

Out-of-plane states The region of existence for the fixed points is divided into two by the sign of M_{eff}. For the easy-plane anisotropy $\nu > 0$,

$$\nu > 1 + \eta_{\mathrm{STT}}^2, \tag{32}$$

$$\nu \lessgtr \left(1 + \frac{2\alpha\eta_{\mathrm{STT}}^2}{\alpha - 2\eta_{\mathrm{STT}} + \alpha\eta_{\mathrm{STT}}^2}\right)\sqrt{\frac{\alpha - 2\eta_{\mathrm{STT}} + \alpha\eta_{\mathrm{STT}}^2}{2\alpha} + \left(\frac{\kappa}{2\alpha\gamma\mu_0 H_{\mathrm{ext}}}\right)^2} + \frac{\kappa}{2\alpha\gamma\mu_0 H_{\mathrm{ext}}}\frac{\alpha - 2\eta_{\mathrm{STT}} - \alpha\eta_{\mathrm{STT}}^2}{\alpha - 2\eta_{\mathrm{STT}} + \alpha\eta_{\mathrm{STT}}^2}. \tag{33}$$

When the anisotropy is of easy-axis type $\nu < 0$,

$$\nu < -1 - \eta_{\mathrm{STT}}^2, \tag{34}$$

$$\nu \lessgtr -\left(1 + \frac{2\alpha\eta_{\mathrm{STT}}^2}{\alpha - 2\eta_{\mathrm{STT}} + \alpha\eta_{\mathrm{STT}}^2}\right)\sqrt{\frac{\alpha - 2\eta_{\mathrm{STT}} + \alpha\eta_{\mathrm{STT}}^2}{2\alpha} + \left(\frac{\kappa}{2\alpha\gamma\mu_0 H_{\mathrm{ext}}}\right)^2} + \frac{\kappa}{2\alpha\gamma\mu_0 H_{\mathrm{ext}}}\frac{\alpha - 2\eta_{\mathrm{STT}} - \alpha\eta_{\mathrm{STT}}^2}{\alpha - 2\eta_{\mathrm{STT}} + \alpha\eta_{\mathrm{STT}}^2}. \tag{35}$$

Note that for $\alpha\eta_{\mathrm{STT}} > 1 - \sqrt{1 - \alpha^2 - \kappa^2/\gamma^2\mu_0^2 H_{\mathrm{ext}}^2}$, the square root becomes imaginary and the inequalities do not make sense. The stability can be determined directly from Eq. (25), nevertheless; they are unstable for $\nu < 0$ and stable for $\nu > 0$.

In specific ranges of η_{STT}, ν, all the fixed points are unstable. The Poincaré-Bendixon's theorem excludes chaos or strange attractors as asymptotic states of the Bloch sphere. Therefore, in that region of parameter space, the attractor state should be a limit cycle referred to as auto-oscillation in magnetism. Although we are unable to exclude limit cycles as possible attractors when some of the fixed points are stable, numerical simulations show no an evidence of asymptotic behaviour other than convergence to a stable fixed point. This allows us to generate a bifurcation diagram that classifies the parameter regions according to their attractors. Figure 4a in the main text is an example for $\alpha = 0.1, \kappa = 0$.

5. Interpretation of the ST-FMR results

The dynamical systems analysis suggests three regimes under our experimental condition $0 < M_{\mathrm{eff}} \lesssim H_{\mathrm{ext}}$, *viz.* $\boldsymbol{M}_0 \parallel \boldsymbol{H}_{\mathrm{ext}}$, $\boldsymbol{M}_0 \parallel -\boldsymbol{H}_{\mathrm{ext}}$, and a bistability between these two. Upon sweeping $I_{\mathrm{dc}} \sin\phi$ from positive to negative values, one should see the parallel, bistable, and anti-parallel states appear in this order. We measure ST-FMR instead at fixed I_{dc} and ω while sweeping H_{ext}. This corresponds to following a straight line passing $\eta_{\mathrm{STT}} = \nu = 0$ in the bifurcation diagram (Fig. 4a in the main text), during which the stability of the fixed points can change. The ST-FMR voltage expression given in Eq. (11) depends on the reference state, namely the upper and lower signs have to be used for the parallel and anti-parallel fixed points respectively. Therefore, within any magnetic field interval in which a stability change occurs, the usual fitting procedure of the voltage would not work. Indeed, at $f = \omega/2\pi = 2.5$ GHz in Fig. 2f in the main text, the absence of clear resonance can be interpreted as H_{res} at 2.5 GHz falling in that interval of stability change for $I_{\mathrm{dc}} = 4$ mA and $\phi = 225°$.

The $\pm$ sign in Eq. (11) suggests that the sign of the resonance peak in V_{dc} discriminates the parallel or anti-parallel fixed points. However, numerous complications exist. The assumption that $\Delta H_{\pm}$ does not change sign as a function of ω and I_{dc} does not hold according to Eq. (13). The field linewidth at the critical current of a stability change of the fixed points at $H_{\mathrm{ext}} = H^{\pm}_{\mathrm{res}}$ reads

$$\Delta H_{\pm} = \Delta H_0 \pm \frac{\alpha\omega}{\gamma\mu_0} \mp \left\{\alpha\left(H^{\pm}_{\mathrm{res}} \pm \frac{M_{\mathrm{eff}}}{2}\right) \pm \frac{\kappa}{\gamma\mu_0}\right\}\sqrt{\frac{1}{1+\gamma^2\mu_0^2 M_{\mathrm{eff}}^2/4\omega^2}} = 0. \tag{36}$$

This ensures that at least at the resonance field, the stability change of the fixed points corresponds to the change of sign of $\Delta H^{\pm}_{\mathrm{res}}$. A fixed point should be stable whenever a clear resonance around it is observable. Therefore, one may assume $\Delta H_{\pm} > 0$ when fitting a resonance peak regardless of the reference state. If ω and I_{dc} were tuned to satisfy Eq. (36) exactly, the stability change between $H_{\mathrm{ext}} \lessgtr H^{\pm}_{\mathrm{res}}$ would prevent a clear Lorentzian signal. Moreover, as discussed in the previous section, V_{dc} is not Lorentzian fwhen $\Delta H_{\pm} \sim 0$ for $H_{\mathrm{ext}} \sim H^{\pm}_{\mathrm{res}}$. Hence, the interpretation of an observed peak as a resonance according to Eq. (11) can be justified only if for the given ω and I_{dc} the stability of the fixed points does not change in the interval $H^{\pm}_{\mathrm{res}} - \Delta H_{\pm} \lesssim H_{\mathrm{ext}} \lesssim H^{\pm}_{\mathrm{res}} + \Delta H_{\pm}$. Only then, a sign change of the resonance peak would be evidence for a magnetisation reversal.

Supplementary Note 6: Fokker-Planck Equation

Thermal excitation induces random fluctuations of the magnetic order by a noise magnetic field $\boldsymbol{\xi}(t)$ (in units of T, *i.e.*, magnetic flux density) in the LLG equation Eq. (1). When terms of order α^2 are disregarded, the stochastic LLG equation in the absence of a microwave drive is equivalent to a Fokker-Planck equation for the probability distribution function $f(\boldsymbol{n},t)$ with $\boldsymbol{n}$ the dimensionless unit vector of the magnetisation[11].

$$\frac{\partial f}{\partial t} = \gamma\mu_0 \frac{\partial}{\partial \boldsymbol{n}} \cdot \left[\boldsymbol{n} \times \left\{\boldsymbol{H}_{\rm ext} - M_{\rm eff} n_z \hat{\boldsymbol{z}} + \boldsymbol{n} \times \left(\alpha \boldsymbol{H}_{\rm ext} - \alpha M_{\rm eff} n_z \hat{\boldsymbol{z}} + \beta I_{\rm dc}\hat{\boldsymbol{y}} - \frac{\alpha k_B T}{\mu_0 M_{\rm s} V_{\rm a}} \frac{\partial}{\partial \boldsymbol{n}}\right)\right\} f\right]. \tag{37}$$

One can solve this equation in the relevant limit of vanishing magnetic anisotropy $M_{\rm eff} = 0$ and an STT spin polarisation parallel to $\boldsymbol{H}_{\rm ext}$ *i.e.*, $\hat{\boldsymbol{y}} \to (\hat{\boldsymbol{y}} \cdot \boldsymbol{H}_{\rm ext})\,\boldsymbol{H}_{\rm ext}/H_{\rm ext}^2 = \boldsymbol{H}_{\rm ext}\sin\phi/H_{\rm ext}$. In the steady state $\partial f/\partial t = 0$:

$$f(\boldsymbol{n}) = \frac{\Delta_{\rm STT}}{4\pi \sinh \Delta_{\rm STT}} \exp\left(\Delta_{\rm STT} \frac{\boldsymbol{H}_{\rm ext}}{H_{\rm ext}} \cdot \boldsymbol{n}\right), \tag{38}$$

where $\Delta_{\rm STT}$ is a thermal stability factor defined by

$$\Delta_{\rm STT} = \frac{\mu_0 M_{\rm s} H_{\rm ext} V_{\rm a}}{k_B T}\left(1 + \frac{\beta I_{\rm dc}\sin\phi}{\alpha H_{\rm ext}}\right). \tag{39}$$

The prefactor outside the curly brackets is the ratio between the Zeeman and thermal energies. f reduces to the Boltzmann distribution in the absence of the STT. A current with $I_{\rm dc}\sin\phi < 0$ decreases $\Delta_{\rm STT}$ until the critical value $\beta I_{\rm dc}\sin\phi = -\alpha H_{\rm ext}$, at which $\Delta_{\rm STT} = 0$ and $f = 1/4\pi$ does not depend on $\boldsymbol{n}$, namely the probability distribution becomes isotropic over the entire Bloch sphere. For larger currents $\beta I_{\rm dc}\sin\phi < -\alpha H_{\rm ext}$, $\Delta_{\rm STT} < 0$, which looks like a negative effective magnetic field that favours the magnetisation vectors in the southern Bloch hemisphere. This is a probabilistic picture of the magnetisation reversal by the STT in the presence of thermal noise.

Using the probability distribution, the first and second moments of the magnetisation components read

$$\begin{pmatrix}\langle M_x\rangle\\ \langle M_y\rangle\\ \langle M_z\rangle\end{pmatrix} = M_{\rm s}\left(\coth\Delta_{\rm STT} - \frac{1}{\Delta_{\rm STT}}\right)\begin{pmatrix}\cos\phi\\ \sin\phi\\ 0\end{pmatrix}, \tag{40}$$

and

$$\begin{pmatrix}\langle M_x^2\rangle\\ \langle M_y^2\rangle\\ \langle M_z^2\rangle\end{pmatrix} = M_{\rm s}^2\begin{pmatrix}\cos^2\phi\\ \sin^2\phi\\ 0\end{pmatrix} + \frac{M_{\rm s}^2}{\Delta_{\rm STT}}\left(\coth\Delta_{\rm STT} - \frac{1}{\Delta_{\rm STT}}\right)\begin{pmatrix}1-3\cos^2\phi\\ 1-3\sin^2\phi\\ 1\end{pmatrix}, \tag{41}$$

where the angled brackets denote the thermal averaging. $\coth\Delta_{\rm STT} - 1/\Delta_{\rm STT} \to \Delta_{\rm STT}/3$ as $\Delta_{\rm STT} \to 0$ confirms the isotropic magnetisation distribution at the critical current. Since AMR $\propto \langle M_x^2\rangle$ and SMR $\propto M_{\rm s}^2 - \langle M_y^2\rangle$ we expect different results for these mechanisms, even for an in-plane angle (ϕ) scan, as can be seen in Figs. 5a-b. The SMR agrees better with the experimental results. $\coth\Delta$ in Eq. (41) indicates an exponential dependence on temperature, and the macrospin volume $V_{\rm a}$. The $I_{\rm dc}$ dependence of the SMR for different values of $V_{\rm a}/T$ in Fig. 5c compared with the rather gradual variation seen in the experiments fixes the order of magnitude of $V_{\rm a}/T$.

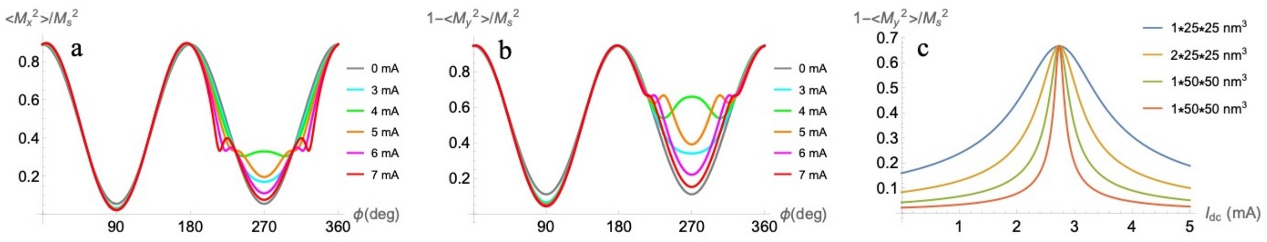

Supplementary Fig. 5. Field angle dependence of AMR (a) and SMR (b) for $\mu_0 H_{\rm ext} = 150$ mT, $V_{\rm a} = 2\times25\times25$ nm^3, and different current values, and current dependence of SMR (c) for $\mu_0 H_{\rm ext} = 100$ mT and different values of $V_{\rm a}$. The other parameters are the same as specified in Methods of the main text.

Supplementary Note 7: First Passage Time

The stochastic dynamics of the magnetisation on the two-dimensional Bloch sphere can be can be characterized by fast precessional motion and slow drift-diffusion in energy, both of which are one-dimensional, if there is a hierarchy between the energy-conserving deterministic torques (*e.g.* the magnetic field and anisotropy) and the damping, STT, and fluctuations that all change the energy[12]. The magnetisation switching rate is then related to the inverse first passage time[13,14]. Here, we apply the same technique to calculate the dwelling time in the regime of dynamical bistability.

We assume $\boldsymbol{H}_{\rm ext}$ and the polarisation of the spin current to be parallel with $\boldsymbol{H}_{\rm ext} = H_{\rm ext}\hat{\boldsymbol{y}}$ and $I_{\rm dc} \longrightarrow I_{\rm dc}\sin\phi$. Our model then reduces to a special case of Newhall and Vanden-Eijnden's work[13] with $\beta_y = 0$, $\beta_z \neq 1/2$, and $x \leftrightarrow y$ in their notation. While they normalised the energy and time by $\mu_0 M_{\rm s}^2 V_{\rm a}$ and $1/\gamma\mu_0 M_{\rm s}$ respectively, it suits us better to normalise them by $\mu_0 M_{\rm s} H_{\rm ext} V_{\rm a}$ and $1/\gamma\mu_0 H_{\rm ext}$, denoted by dimensionless variables ϵ and τ. The stochastic equation after averaging of the stochastic LLG over the fast energy-conserving dynamics is

$$\frac{d\epsilon}{d\tau} = -\alpha A(\epsilon) - \eta_{\rm STT} B(\epsilon) + \frac{2\alpha}{\Delta} C(\epsilon) + \sqrt{\frac{2\alpha}{\Delta} A(\epsilon)}\zeta(\tau), \tag{42}$$

where $\eta_{\rm STT} = \beta I_{\rm dc}\sin\phi / H_{\rm ext}, \Delta = \mu_0 M_{\rm s} H_{\rm ext} V_{\rm a}/k_B T$, $\zeta(\tau)$ is the normalised white noise satisfying $\langle\zeta(\tau)\zeta(\tau')\rangle = \delta(\tau-\tau')$, and the coefficients read

$$A(\epsilon) = \nu^2\frac{\overline{M_z^2}}{M_{\rm s}^2} - 4\epsilon^2 - 4\epsilon\frac{\overline{M_y}}{M_{\rm s}} + 1 - \frac{\overline{M_y^2}}{M_{\rm s}^2}, \tag{43}$$

$$B(\epsilon) = 2\epsilon\frac{\overline{M_y}}{M_{\rm s}} + 1 + \frac{\overline{M_y^2}}{M_{\rm s}^2}, \tag{44}$$

$$C(\epsilon) = \frac{\nu}{2} - 3\epsilon - 2\frac{\overline{M_y}}{M_{\rm s}}. \tag{45}$$

We do not have to worry about different branches[13] since the energy fixes the orbits unambiguously. The overbars denote averaging over a cycle of the energy-conserving fast dynamics:

$$\frac{\overline{M_y}}{M_{\rm s}} = \frac{1}{\tau_\circ\nu}\int_0^{2\pi}\frac{\sqrt{1-2\epsilon\nu\sin^2\phi+\nu^2\sin^4\phi}-1}{\sin^2\phi+\left(\sqrt{1-2\epsilon\nu\sin^2\phi+\nu^2\sin^4\phi}-1\right)\cos^2\phi}d\phi, \tag{46}$$

$$\frac{\overline{M_y^2}}{M_{\rm s}^2} = \frac{1}{\tau_\circ\nu^2}\int_0^{2\pi}\frac{\left(\sqrt{1-2\epsilon\nu\sin^2\phi+\nu^2\sin^4\phi}-1\right)^2}{\sin^2\phi+\left(\sqrt{1-2\epsilon\nu\sin^2\phi+\nu^2\sin^4\phi}-1\right)\cos^2\phi}\frac{d\phi}{\sin^2\phi}, \tag{47}$$

$$\frac{\overline{M_z^2}}{M_{\rm s}^2} = \frac{1}{\tau_\circ\nu^2}\int_0^{2\pi}\frac{\nu^2\sin^4\phi-\left(\sqrt{1-2\epsilon\nu\sin^2\phi+\nu^2\sin^4\phi}-1\right)^2}{\sin^2\phi+\left(\sqrt{1-2\epsilon\nu\sin^2\phi+\nu^2\sin^4\phi}-1\right)\cos^2\phi}d\phi, \tag{48}$$

with period

$$\tau_\circ = \int_0^{2\pi}\frac{\sin^2\phi}{\sin^2\phi+\left(\sqrt{1-2\epsilon\nu\sin^2\phi+\nu^2\sin^4\phi}-1\right)\cos^2\phi}d\phi. \tag{49}$$

According to Feller[15], the first passage time $\tau(x;\epsilon_1,\epsilon_2)$ from $x \in (\epsilon_1,\epsilon_2)$ to one of the boundaries $x = \epsilon_1$ or $x = \epsilon_2$ obeys

$$\left\{-\alpha A(x) - \eta_{\rm STT} B(x) + \frac{2\alpha}{\Delta}C(x)\right\}\frac{d\tau}{dx} + \frac{\alpha}{\Delta}A(x)\frac{d^2\tau}{dx^2} = -1; \tag{50}$$

The absorbing boundary conditions $\tau(\epsilon_1) = \tau(\epsilon_2) = 0$ make sense when both ϵ_1, ϵ_2 are accessible boundaries. However, we are interested in the passage to and from the north and south poles $x = \mp 1$, which were claimed to be entrance boundary points[13]

with $A(\pm 1) = 0$, and unreachable from any interior point $x \in (-1, 1)$ in a finite time. However, we first show that $\epsilon = \pm 1$ are in fact accessible boundary points at least in our model with $0 < |\nu| < 1$ as follows. Let $F(x)$ be an indefinite integral defined by

$$F(x) = \Delta \int^{x} \left\{ -1 - \frac{\eta_{\mathrm{STT}}}{\alpha} \frac{B(y)}{A(y)} + \frac{2}{\Delta} \frac{C(y)}{A(y)} \right\} dy. \tag{51}$$

A boundary point x_0 is accessible if the functions $e^{-F(x)}$ and $A(x)^{-1} e^{F(x)}$ are both integrable in some neighbourhood of x_0[15]. One may numerically check that $A(x), B(x), C(x)$ are all smooth functions in an open interval containing $[-1, 1]$ with $x = \pm 1$ the only zeros of $A(x)$ (Fig. 6). Therefore, we just need to check the integrability around $x_0 = \pm 1$. The integrals Eqs. (46) - (49) and their derivatives can be analytically evaluated for $\epsilon = \pm 1$ as $A(\pm 1) = 0, B(\pm 1) = 0, C(\pm 1) = \mp (2 \mp \nu)/2$ and

$$A(\epsilon) = \left(2 \mp \nu + \frac{\nu^2}{2 \mp \nu}\right)(1 \mp \epsilon) + o(1 \mp \epsilon), \tag{52}$$

$$B(\epsilon) = 2(1 \mp \epsilon) + o(1 \mp \epsilon). \tag{53}$$

Thus one obtains

$$F(x) = \ln (1 \mp x)^{a} + \text{regular terms.}, \quad a = \left\{ 1 + \left(\frac{\nu}{2 \mp \nu} \right)^2 \right\}^{-1} < 1. \tag{54}$$

Therefore, $e^{-F(x)} \sim (1 \mp x)^{-a}$ and $A(x)^{-1} e^{F(x)} \sim (1 \mp x)^{a-1}$ around $x = \pm 1$, suggesting they are integrable.

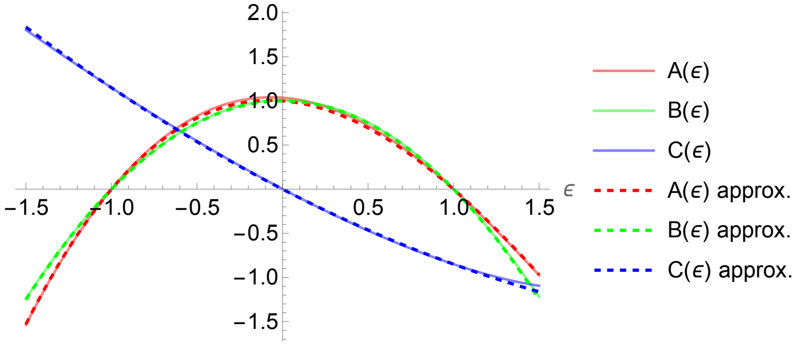

Supplementary Fig. 6. The coefficient functions Eqs. (43) - (45) (solid lines) and their approximations Eqs. (59) - (61) (dashed lines) for $\nu = M_{\mathrm{eff}}/H_{\mathrm{ext}} = 0.3$.

Once the poles are accessible in a finite time, some boundary conditions other than $\tau(\pm 1) = 0$ have to be imposed to describe how the magnetisation returns back from the boundaries. While the natural choice is the reflecting lateral conditions[15], we avoid this consideration by taking advantage of our interest in the small ν regime. Namely, the integrals Eqs. (46) - (49) can be approximately computed as

$$\tau_{\circ} = \frac{2\pi}{\sqrt{1 - \epsilon\nu}} + O\left(\nu^2\right), \tag{55}$$

$$\frac{\overline{M_y}}{M_s} = -\epsilon + \frac{1 - \epsilon^2}{4}\nu + O\left(\nu^2\right), \tag{56}$$

$$\frac{\overline{M_y^2}}{M_s^2} = \epsilon^2 - \frac{1 - \epsilon^2}{2}\epsilon\nu + O\left(\nu^2\right), \tag{57}$$

$$\frac{\overline{M_z^2}}{M_s^2} = \frac{1 - \epsilon^2}{2}\left(1 + \frac{\epsilon\nu}{2}\right) + O\left(\nu^2\right). \tag{58}$$

Therefore the coefficient functions in Eqs. (43) - (45) read

$$A(\epsilon) \approx \left(1 - \epsilon^2\right)\left(1 - \frac{\epsilon\nu}{2}\right), \tag{59}$$

$$B(\epsilon) \approx 1 - \epsilon^2, \tag{60}$$

$$C(\epsilon) \approx -\epsilon + \frac{\nu\epsilon^2}{2}. \tag{61}$$

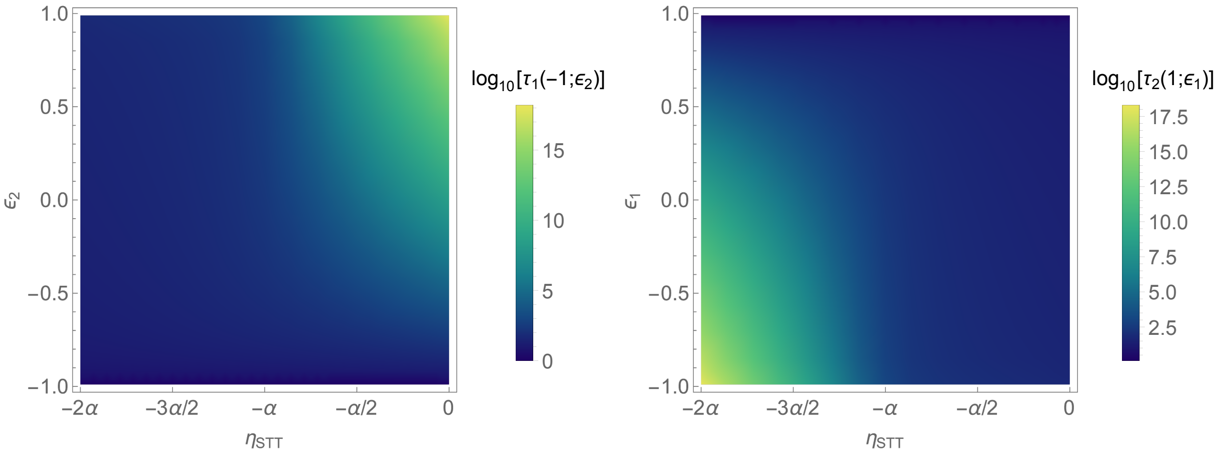

Supplementary Fig. 7. First passage times for $\alpha = 0.09, \nu = 0.2$ from south to north $\lim_{x\to-1} \tau_1(x;\epsilon_2)$ (left panel) and north to south $\lim_{x\to+1}(x;\epsilon_1)$ (right panel) as functions of $\eta_{\rm STT}$ and the end point *i.e.*, ϵ_2 for τ_1 and ϵ_1 for τ_2.

As can be seen in Fig. 6, the difference from the exact result is barely noticeable and we adopt the approximated equation as our physical model. At this first order in ν, it turns out that $e^{-F(x)}$, constructed from the approximate coefficient functions, is not integrable, which implies the boundaries $x = \pm 1$ are inaccessible. One can then expect the following:

1. If one of $\epsilon_1 \to -1$ or $\epsilon_2 \to 1$ is taken, it is expected that the probability of the system reaching that boundary goes to zero and $\tau(x;\epsilon_1,\epsilon_2)$ should give the first passage time from x to $\epsilon_2 < 1$ or $\epsilon_1 > -1$ accordingly.

2. If both $\epsilon_1 \to -1, \epsilon_2 \to 1$ are taken, $\tau(x) \to \infty$ is expected for every x.

Therefore, in order to estimate the switching time from the north pole to the south and *vice versa*, we calculate $\tau_1(x;\epsilon_2) \equiv \lim_{\epsilon_1\to-1} \tau(x;\epsilon_1,\epsilon_2)$ and $\tau_2(x;\epsilon_1) \equiv \lim_{\epsilon_2\to1} \tau(x;\epsilon_1,\epsilon_2)$. We then take the limit $x \to -1$ for an arbitrarily $\epsilon_2 \lesssim 1$ and $x \to 1$ for an arbitrarily $\epsilon_1 \gtrsim -1$. For this programme, we first rewrite Eq. (50) as

$$\frac{d}{dx}\left\{e^{F(x)}\frac{d\tau}{dx}\right\} = -\frac{\Delta e^{F(x)}}{\alpha A(x)}. \tag{62}$$

The integration constant of F cancels in Eq. (62), and the right-hand-side is integrable. When solving Eq. (62), the integration constants do have to be specified such that the boundary conditions $\tau(\epsilon_1) = \tau(\epsilon_2) = 0$ are satisfied. Since we eventually take $\epsilon_1 \to -1$ for τ_1 and $\epsilon_2 \to 1$ for τ_2 respectively, it is more convenient to make different choices between the two

$$\tau_1(x;\epsilon_2) = \int_x^{\epsilon_2} e^{-F(y)}\{G_1(y) - C_1\}\,dy, \quad G_1(y) = -\frac{\Delta}{\alpha}\int_y^{\epsilon_2}\frac{e^{F(z)}}{A(z)}dz, \tag{63}$$

$$\tau_2(x;\epsilon_1) = \int_{\epsilon_1}^{x} e^{-F(y)}\{G_2(y) - C_2\}\,dy, \quad G_2(y) = -\frac{\Delta}{\alpha}\int_{\epsilon_1}^{y}\frac{e^{F(z)}}{A(z)}dz, \tag{64}$$

which automatically satisfy $\tau_1(\epsilon_2;\epsilon_2) = \tau_2(\epsilon_1;\epsilon_1) = 0$ and the dependence on ϵ_1 for τ_1 or ϵ_2 for τ_2 is contained entirely in C_1 or C_2 respectively. Note that $G_1(y), G_2(y)$ are bounded in $[-1,1]$. The remaining integration constants C_1, C_2 are chosen so as to satisfy $\tau_1(\epsilon_1;\epsilon_2) = \tau_2(\epsilon_2;\epsilon_1) = 0$, yielding

$$C_1(\epsilon_1) = \int_{\epsilon_1}^{\epsilon_2} e^{-F(y)}G_1(y)\,dy \bigg/ \int_{\epsilon_1}^{\epsilon_2} e^{-F(y)}dy, \tag{65}$$

$$C_2(\epsilon_2) = \int_{\epsilon_1}^{\epsilon_2} e^{-F(y)}G_2(y)\,dy \bigg/ \int_{\epsilon_1}^{\epsilon_2} e^{-F(y)}dy. \tag{66}$$

When the limit $\epsilon_1 \to -1$ for C_1 or $\epsilon_2 \to 1$ for C_2 is taken, the integrals both in the numerator and denominator will be dominated by the region $y \approx -1$ or $y \approx 1$ respectively, as $e^{-F(y)} \sim (1 \pm y)^{-1}$. Thus, one can guess that $\lim_{\epsilon_1\to-1} C_1(\epsilon_1) = G_1(-1)$ and

$\lim_{\epsilon_2 \to 1} C_2(\epsilon_2) = G_2(1)$. We accept them without a proof and obtain

$$\tau_1(x;\epsilon_2) = \frac{\Delta}{\alpha}\int_x^{\epsilon_2} e^{-F(y)}\left\{\int_{-1}^{y}\frac{e^{F(z)}}{A(z)}dz\right\}dy \approx \frac{\Delta}{\alpha}\int_x^{\epsilon_2}\int_{-1}^{y}\frac{1}{1-y^2}\left(\frac{2-\nu z}{2-\nu y}\right)^{\frac{2\eta_{\mathrm{STT}}\Delta}{\alpha\nu}}\left(1-\frac{\nu z}{2}\right)^{-1}e^{\Delta(y-z)}dzdy, \quad (67)$$

$$\tau_2(x;\epsilon_1) = \frac{\Delta}{\alpha}\int_{\epsilon_1}^{x} e^{-F(y)}\left\{\int_{y}^{1}\frac{e^{F(z)}}{A(z)}dz\right\}dy \approx \frac{\Delta}{\alpha}\int_{\epsilon_1}^{x}\int_{y}^{1}\frac{1}{1-y^2}\left(\frac{2-\nu z}{2-\nu y}\right)^{\frac{2\eta_{\mathrm{STT}}\Delta}{\alpha\nu}}\left(1-\frac{\nu z}{2}\right)^{-1}e^{\Delta(y-z)}dzdy. \quad (68)$$

We remark that although the argument about the limits of C_1, C_2 does not hold when the boundaries are accessible, the expressions Eqs. (67) and (68) before substituting the approximations in fact agree with what they should be under the reflecting lateral conditions.

The integrals are evaluated and plotted in Fig. 7. The anisotropy $\nu = 0.2$ models $\mu_0 M_{\mathrm{eff}} = 30$ mT and $\mu_0 H_{\mathrm{ext}} = 150$ mT. To compare the result with the dwelling time observed in Fig. 4e in the main text, we note that the first passage times are $\tau_1 \sim \tau_2 \sim 10^3$ for $\eta_{\mathrm{STT}} = -\alpha$. For $\mu_0 H_{\mathrm{ext}} = 150$ mT, $1/\gamma\mu_0 H_{\mathrm{ext}} = 0.24$ ns so that the theory predicts a dwelling time of order microseconds in a qualitative agreement with the stochastic simulation.

Supplementary Note 8: Joule Heating

We include the effect of Joule heating by a temperature-dependent anisotropy and magnetisation. The magnetisation of W|CoFeB(1.3 nm)|MgO in Fig. 2b of Ref. 16 is well represented by Kuz'min's equation[17]:

$$m(T) = \left(1 - s(T/T_\mathrm{C})^{3/2} - (1-s)(T/T_\mathrm{C})^p\right)^{1/3} \tag{69}$$

with $p = 2.5$, $s = 2.5$ and $T_\mathrm{C} = 833$ K. The interface anisotropy scales as $K(T)/K(0) = (M(T)/M(0))^2$ according to the Callen-Callen theory[18]. The magnetisation and anisotropy at a given temperature are:

$$M_\mathrm{s}(T) = M_\mathrm{s}\frac{m(T/T_\mathrm{C})}{m(300K)} \quad K(T) = K\frac{m(T/T_\mathrm{C})^2}{m(300K)^2}. \tag{70}$$

Using the electrical resistance as a temperature sensor, we estimate $\Delta T_\mathrm{max} = 100$ K for the maximum current $j_\mathrm{max} = 8$ mA and interpolate for the temperature increase as

$$T = T_\mathrm{ambient} + \Delta T_\mathrm{max}\left(\frac{j}{j_\mathrm{max}}\right)^2. \tag{71}$$

Supplementary Note 9: Continuous restricted Boltzmann machines using nearly isotropic magnets

Here we show probabilistic computing applications using the stochasticity of an array of nearly-isotropic magnets as a computational resource. Electrically controlled zero damping states achieved in our study represent a platform to construct probabilistic bits (p-bits) due to its stochastic transitions across the entire Bloch sphere. Our p-bits can output a continuous variable between the two states, i.e. $x \in [-1, 1]$. Such behaviour stands in stark contrast to that of binary p-bits ($x \in \{-1, 1\}$), which are based on stochastic magnetic tunnel junctions (s-MTJs) and can be correlated to minimise an Ising Hamiltonian[19,20] for binary tasks such as machine learning[21], combinatorial optimisation[19,21,22] and quantum emulation[21,23]. P-bits can also be used for the Boltzmann machine (BM), where one can make use of the BM update rules[24] to train the synaptic connections between p-bits to sample from desired distributions.

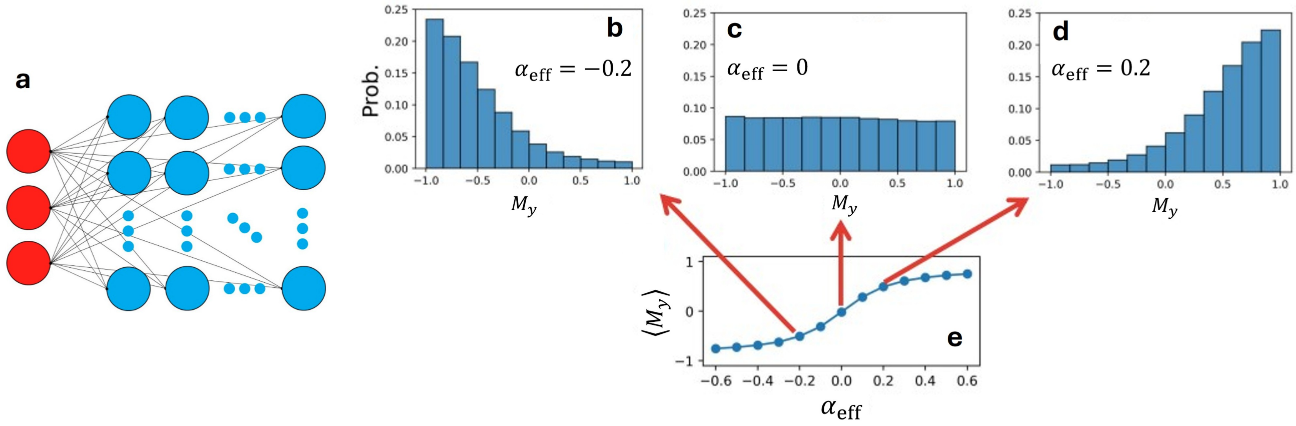

Supplementary Fig. 8. a, Schematic of a RBM. Hidden units (red) are synaptically connected to visible units (blue). **b-d,** Histograms showing M_y values for different effective damping parameters. **e,** Time-averaged M_y for varied $\alpha_{\rm eff}$. Simulations were performed using the Euler method with the following parameters: time-step 0.1 ps (made under the assumption of a 100mT external field), total simulation time 1 μs, $V_a = 1 \times 4 \times 4$ nm^3, $\alpha = 0.1$ and $M_{\rm eff}/H_{\rm ext} = 0$.

Restricted Boltzmann machines (RBMs) offer an effective architecture to generate binary data[25,26]. RBMs comprise two groups of binary stochastic variables $\{0, 1\}$: *visible* p-bits ($\mathbf{v}$) and *hidden* p-bits ($\mathbf{h}$) that are connected by weights ($\mathbf{W}$), as schematically shown in Fig. 8a. Both layers are also influenced by visible and hidden bias vectors, $\boldsymbol{\chi}$ and $\boldsymbol{\rho}$ respectively. The Boltzmann distribution for this network can therefore be expressed as[26]:

$$p(\mathbf{v}, \mathbf{h}) = \frac{1}{Z} \mathrm{e}^{-H(\mathbf{v}, \mathbf{h})} \tag{72}$$

where Z is the partition function and the Hamiltonian, $H(\mathbf{v}, \mathbf{h})$, is defined as:

$$H(\mathbf{v}, \mathbf{h}) = -\sum_{ij} v_i W_{ij} h_j - \sum_i \chi_i v_i - \sum_j \rho_j h_j \tag{73}$$

The binary magnetisation state (m) of an s-MTJ at time t is represented by[27]:

$$m(t) = \text{sign}[\text{rand(-1,1)}+\tanh[I(t)]] \tag{74}$$

where rand(-1,1) is a uniform random number between -1 and 1, and $I(t)$ is the input current which can bias the s-MTJ state into either configuration via spin transfer torque. The training procedure for a RBM uses Gibbs Sampling as follows[24]:

1. Randomly initialise the weight matrix according to $W \sim \mathcal{N}(0, 0.1)$ and set the bias vectors, $\boldsymbol{\chi}$ and $\boldsymbol{\rho}$, to zero.
2. Input a data sample to the visible layer by clamping the visible units, $\mathbf{v}^0$, to a data point (e.g. an image from the Fashion MNIST dataset).

3. Sample the hidden units using the conditional probability: $\mathbf{h}^{(0)} \sim P(\mathbf{h}^{(0)} = 1|\mathbf{v}^{(0)})$.
4. Sample the visible units using the conditional probability: $\mathbf{v}^{(1)} \sim P(\mathbf{v}^{(1)} = 1|\mathbf{h}^{(0)})$.
5. Sample the hidden units using the conditional probability: $\mathbf{h}^{(1)} \sim P(\mathbf{h}^{(1)} = 1|\mathbf{v}^{(1)})$.
6. Calculate the weight and bias updates using contrastive divergence: $\Delta\mathbf{W} = \eta[\mathbf{v}^{(0)}\mathbf{h}^{(0)\mathrm{T}} - \mathbf{v}^{(1)}\mathbf{h}^{(1)\mathrm{T}}]$, $\Delta\boldsymbol{\chi} = \eta[\mathbf{v}^{(0)} - \mathbf{v}^{(1)}]$ and $\Delta\boldsymbol{\rho} = \eta[\mathbf{h}^{(0)} - \mathbf{h}^{(1)}]$, where η is the learning rate hyperparameter.
7. Apply the updates to the weight matrix and bias vectors.
8. Repeat steps 2-7 for each data point in the training data batch and monitor the model's performance using the metrics shown in Table 1.

Inference is subsequently performed using the optimised model parameters (with initially randomised visible and hidden units) to repeat steps 3 and 4, N times, to generate new data points. It is possible to amend this binary model to output continuous data by taking an average value of each visible p-bit, yielding values on the interval [0,1]. This augmentation is termed a rate-coded RBM (RBMrate)[28,29], and can be physically modelled using established s-MTJ p-bits[21]. However, RBMrate models are limited in their implementation as repetitive sampling is unfeasible in hardware for large p-circuits[28,29]. As well as this, RBMrate is intrinsically prone to mode collapse in their generated samples, as averaging across many binary samples for each continuous output, leads to a loss in data diversity[28].

In order to process continuous data effectively, one must replace the binary operation of the model architecture with a continuous functionality, such that all p-bits exist on the interval [0,1] at every timestep. This architecture is known as a continuous restricted Boltzmann machine (cRBM) and has been extensively researched using software approaches[28–31]. A cRBM fundamentally differs from an RBM/RBMrate by replacing the binary stochastic unit [described by Eq.(74)] with a continuous stochastic unit, where the state of the jth unit, s_j, is defined by[28]:

$$s_j = \varphi\Big[\sum_i W_{ij} s_i + \sigma \times N_j(0,1)\Big] \tag{75}$$

φ is a sigmoid function and $\sigma \times N_j(0,1)$ is a Gaussian sample with standard deviation σ. To physically realise this continuous stochastic unit using the MgO|CoFeB|W stack, we operate in the isotropic limit, i.e. $M_{\mathrm{eff}}/H_{\mathrm{ext}} \to 0$, allowing the system to evolve as a random walk (following Eq. 42) about the axis parallel to the external field (y-axis) at finite temperatures. We can bias the system by tuning the effective damping: $\alpha_{\mathrm{eff}} = \alpha - \beta I_{\mathrm{dc}} \sin\phi / H_{\mathrm{ext}}$ where $\beta I_{\mathrm{dc}} \sin\phi / H_{\mathrm{ext}}$ is the field normalised strength of the spin-current induced anti-damping torque. Figures 8b-d represent the probability distribution of $<M_y>$ for different α_{eff}, with the α_{eff} dependence of its time-average over 1 μs shown in Fig. 8e.

We show the applicability of simulated isotropic MgO|CoFeB|W stacks as visible units in a cRBM for generating continuous data on the Fashion-MNIST benchmark task at inference (Fig. 9a), where α_{eff} serves as the input parameter, after training on an idealised software cRBM architecture. This training process follows the same procedure as the RBM (steps 1-8), except the conditional distributions for the visible units instead take the form $P(\mathbf{v}^{(t_0)}|\mathbf{h}^{(t_0)})$, for an arbitrary timestep t_0. We compare the results of RBMrate and cRBM architectures with identical parameters. Visually, one can see a greater variety in the generated "t-shirt" samples using the cRBM (Fig. 9c) compared to the RBMrate (Fig. 9b). Quantitatively, we use various metrics to measure the quality and diversity of generated samples from the cRBM and RBMrate: the Fréchet Distance (FD)[32], the Multi-Scale Structure Similarity Index (MS-SSIM)[33], the Jensen Shannon Divergence (JSD)[34] and the Number of Statistically Different Bins (NDB)[35]. The FD is a measure of similarity between two distributions that assumes both the real and generated sample sets can be accurately modelled as multivariate Gaussian distributions[32,36]. For real images ($\mathbb{P}_r$) and generated images ($\mathbb{P}_g$) with means μ_r and μ_g and covariances Σ_r and Σ_g respectively, the FD is defined as[32]:

$$\mathrm{FD}(\mathbb{P}_r, \mathbb{P}_g) = |\mu_r - \mu_g|^2 + \mathrm{Tr}\Big(\Sigma_r + \Sigma_g - 2\sqrt{\Sigma_r \Sigma_g}\Big) \tag{76}$$

A lower FD score suggests that the generated data more closely resembles the true data distribution[36]. The SSIM accesses the difference in luminance, contrast and structure between two sets of image samples[37], $\mathbf{x}$ and $\mathbf{y}$. These three comparison components are defined as[38]:

$$l(\mathbf{x}, \mathbf{y}) = \frac{2\mu_x \mu_y + C_1}{\mu_x^2 + \mu_y^2 + C_1} \tag{77}$$

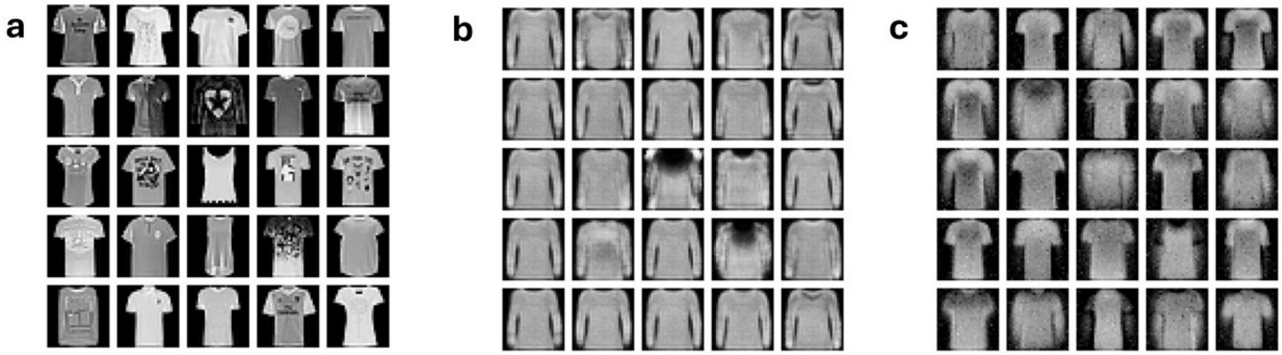

Supplementary Fig. 9. **a,** Sample of the Fashion MNIST training data (t-shirt class). **b & c,** Samples of generated data using RBMrate and cRBM architectures respectively. Both models comprised 10000 binary hidden p-bits and 784 binary/continuous visible units. Idealised models were trained for 5000 epochs to optimise respective model parameters. At inference, the same sLLG parameters were used for the cRBM as the data in Fig. 8, except for $V_a = 1 \times 64 \times 64$ nm^3, to reduce thermal noise. Note that these figures in Fig. 9 are identical to Fig. 5 in the main manuscript.

$$c(\mathbf{x}, \mathbf{y}) = \frac{2\sigma_x\sigma_y + C_2}{\sigma_x^2 + \sigma_y^2 + C_2} \tag{78}$$

$$s(\mathbf{x}, \mathbf{y}) = \frac{\sigma_{xy} + C_3}{\sigma_x\sigma_y + C_3} \tag{79}$$

where μ_x and μ_y are the mean pixel values of **x** and **y**, while σ_x and σ_y are the corresponding standard deviations and σ_{xy} is the covariance. Also, $C_{1,2,3}$ are small stability constants to avoid division by zero. The SSIM is therefore defined as a product of these three components:

$$\text{SSIM}(\mathbf{x}, \mathbf{y}) = l(\mathbf{x}, \mathbf{y}) \cdot c(\mathbf{x}, \mathbf{y}) \cdot s(\mathbf{x}, \mathbf{y}) \tag{80}$$

The MS-SSIM calculates the SSIM at multiple resolutions to capture fine and coarse structures and is defined as[33,37,38]:

$$\text{MS-SSIM}(\mathbf{x},\mathbf{y}) = [l_M(\mathbf{x},\mathbf{y})]^{\alpha_M} \prod_{j=1}^{M} [c_j(\mathbf{x},\mathbf{y})]^{\beta_j} [s_j(\mathbf{x},\mathbf{y})]^{\gamma_j} \tag{81}$$

where the SSIM is evaluated at M different scales and α, β, γ are chosen weight exponents. A perfect MS-SSIM score of 1, indicates that the two sample sets are identical, while a score of 0 suggests that there is no correlation between the sample sets.

	RBMrate	cRBM
FD (*inter*) ↓	0.05	**0.02**
MS-SSIM (*inter*) ↑	0.18	**0.22**
MS-SSIM (*intra*) ↓	0.54	**0.26**
JSD (*inter*) ↓	0.99	**0.90**
JSD (*intra*) ↑	0.33	**0.67**
NDB/*k* (*inter*) ↓	0.97	**0.87**

Table 1. Performances of RBMrate and cRBM on Fashion-MNIST t-shirt generation. 1000 samples were used for every sample set when calculating each metric. Bold values correspond to the best scores and the arrows show whether a higher (↑) or lower (↓) value is optimal for each metric. $k = 30$ was used when calculating NDB/k.

The JSD metric is extracted using feature extraction from principal component analysis (PCA) to broadcast both sets of samples to a 2D feature space. We then partition the feature space into discrete bins, before calculating the JSD using the following expression[34]:

$$\mathrm{JSD}(\mathbf{x} \parallel \mathbf{y}) = \frac{1}{2} D_{\mathrm{KL}}(\mathbf{x} \parallel \mathbf{m}) + \frac{1}{2} D_{\mathrm{KL}}(\mathbf{y} \parallel \mathbf{m}) \tag{82}$$

where $\mathbf{m} = \frac{1}{2}(\mathbf{x} + \mathbf{y})$ and the Kullback Leibler divergence is defined as: $D_{\mathrm{KL}}(\mathbf{p} \parallel \mathbf{q}) = \sum_i p_i \log\left(\frac{p_i}{q_i}\right)$. This provides a similarity measure between two distributions on the interval [0,1], where a lower score indicates that both distributions are closely related[36]. The NDB is a diversity and coverage metric for the generated data which quantifies how well the generated data matches the mode distribution of the real data[36]. We initially utilise k-means clustering on the real data to identify k clusters in the feature space. We then inspect how the generated data is distributed across these k clusters, where we proceed to perform a statistical "z-test" to identify the number of statistically different clusters (bins). Practically, the NDB evaluates the diversity of the generated data by returning the number of image types that our model cannot generate. We report a normalised NDB (NDB/k), where a lower score implies our model is capable of generating more diverse data[35].

It is clear from Table 1 that the cRBM is capable of generating samples which more closely resemble the real data and has a better mode coverage. We use MS-SSIM and JSD in two contexts: *inter* and *intra* where we compare a generated sample set with the real data and two generated sample sets, respectively. We only calculate the FD and NDB/k for the *inter* case, as they are not meaningful metrics for comparing data from the same origin. The cRBM's lower FD score suggests that our generated samples are more structurally similar to the real data and more diverse, compared to the RBMrate's generated data. The MS-SSIM results support this picture, as the cRBM's higher *inter* score implies a better perceptual agreement with the real data and the lower *intra* score indicates a greater diversity. This is reflected in the JSD scores, where the lower *inter* score shows a greater similarity between the real data and the generated data while the higher *intra* score suggests the cRBM has a better mode coverage. Finally, the cRBM's lower NDB/k score shows that the cRBM is able to produce samples that match the real distribution across more modes.

In summary, the cRBM architecture using the isotropic magnets consistently performs better for every metric investigated and is capable of generating more realistic and diverse image data than the RBMrate. The explanation for this stems from two key considerations. Firstly, the cRBM's visible units are continuous, which enable the model to process data using floating point numbers at a higher fidelity than the RBMrate's binary units[31]. Secondly, generating continuous outputs from an RBM necessitates averaging each visible p-bit over a time interval. This leads to a loss in the generated data diversity as the nuances in each binary image are integrated over to output an "averaged" image for every generated sample. The cRBM circumvents this issue as each visible unit exists in a continuous state at every time-step. These findings present a path to physically realise a cRBM using a magnetic system, which cannot be implemented with current binary s-MTJ technology.

Further Supplementary Figures

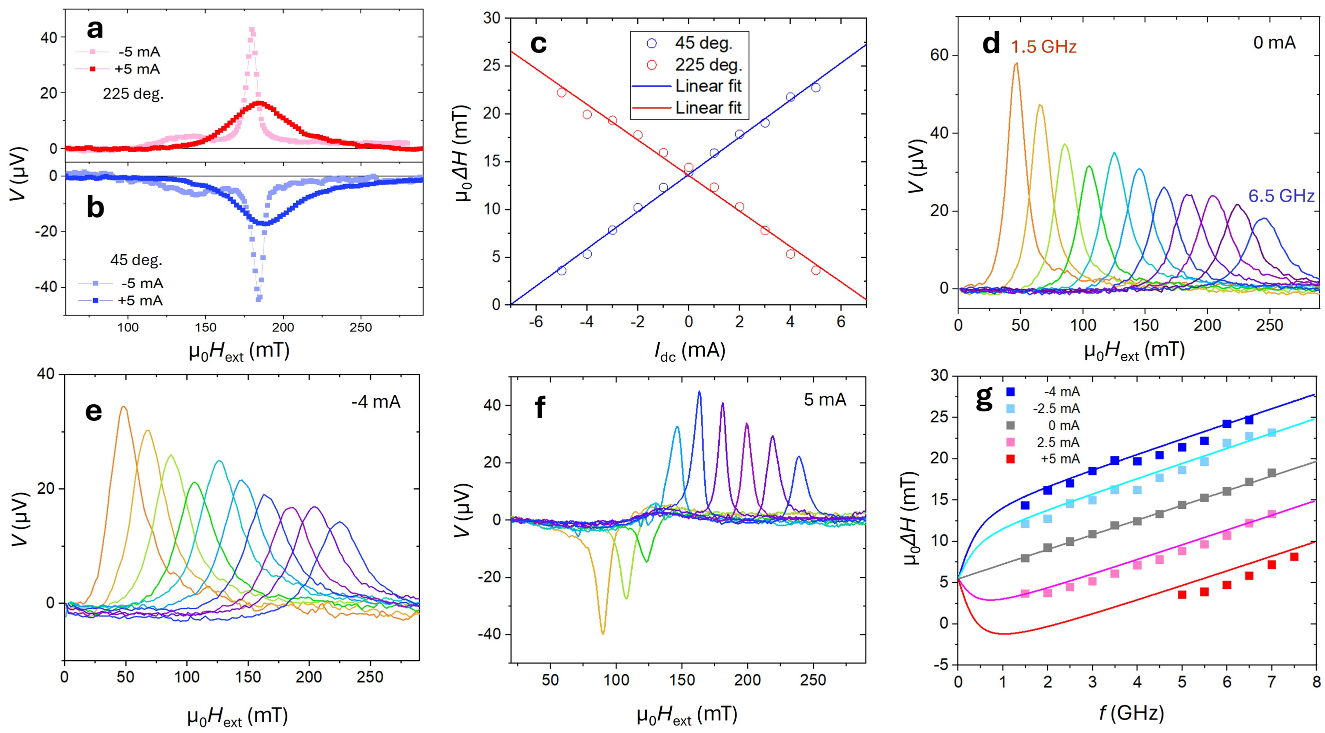

Supplementary Fig. 10. FMR results from another device in a different chip: **a-b**, Field-swept FMR voltages measured for (**a**) $\phi = 225$ deg. and (**b**) $\phi = 45$ deg. **c**, Linewidth extracted from 5 GHz FMR measurements for $\phi = 45$ deg. (blue) and $\phi = 225$ deg.(red), for different I_c values. Best fit linear lines for each measurement set are also shown. **d-f**, Field-swept FMR voltages measured for various frequencies and three different dc current conditions (**d** 0 mA, **e** -4 mA and **f** 5 mA). Magnetic fields were applied along $\phi = 225$ deg. **g**, Linewidth as a function of frequency for different I_{dc}. The grey line is generated by a best linear fit parameter and the rest are calculated by using Eq.(3) in the main manuscript. Extracted physical parameters are $\theta_{SH} = 0.21$, $\beta = 2.5 \times 10^6$ m^{-1}, $\mu_0 M_{eff} = 41.6$ mT, $\alpha = 0.045$ and $\mu_0 \Delta H_0 = 5.5$ mT.

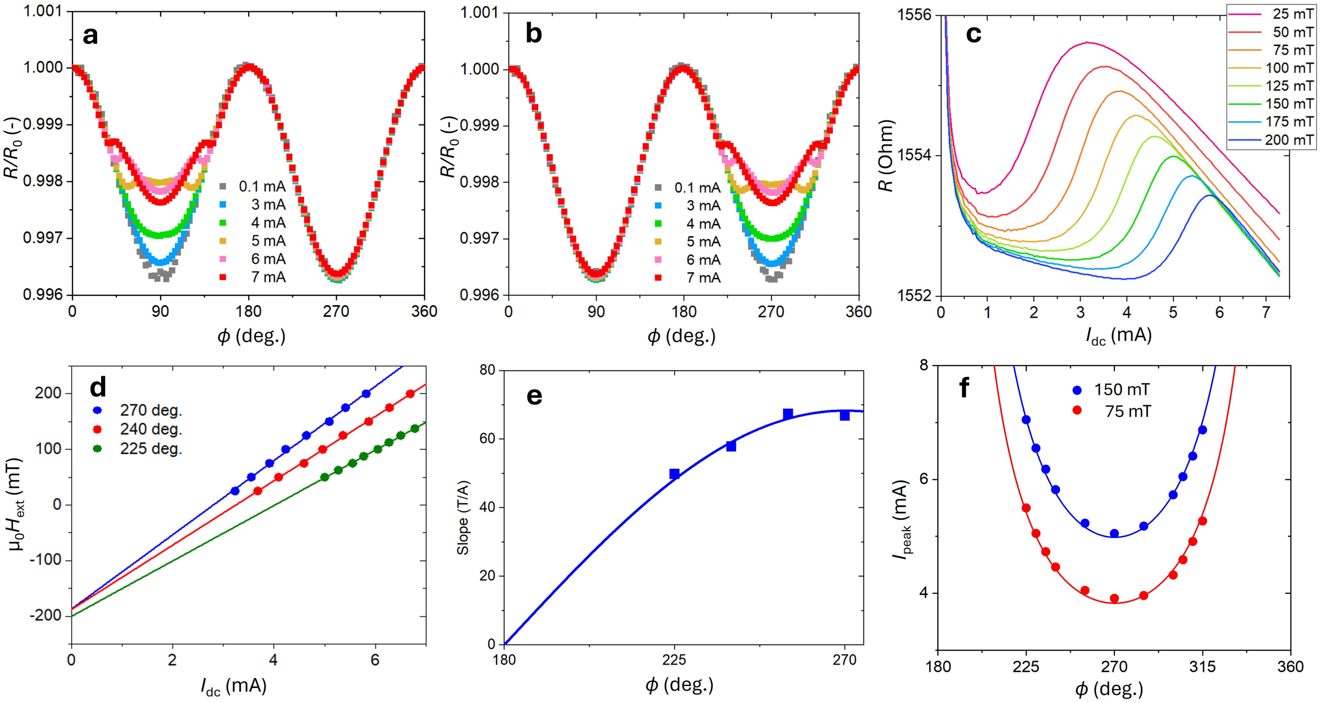

Supplementary Fig. 11. MR results from another device in a different chip: **a-b**, Angular dependence of magnetoresistance measured with different current biases. We applied 150 mT for all the measurements. **c**, Resistance vs dc current measurements at different applied magnetic fields 25-200 mT along $\phi = 270$ deg. **d**, Peak current position measured for different magnetic fields and ϕ, together with best-fit linear lines for each ϕ. **e**, The slope extracted for different ϕ fit using a $\sin\phi$ function. **f**, Peak current position (I_{peak}) as a function of ϕ measured at fixed field values (75 and 150 mT) with curves calculated by Eq. (1) in the main manuscript. The best fit curves lead to $\theta_{\text{SH}}/\alpha = 4.6$; with $\alpha = 0.045$ extracted above, $\theta_{\text{SH}} = 0.21$ is calculated and agrees very well with that quantified by FMR experiments; $\mu_0\Delta H_0' = 8.0$ mT is compared well with $\mu_0\Delta H_0 = 5.5$ mT.

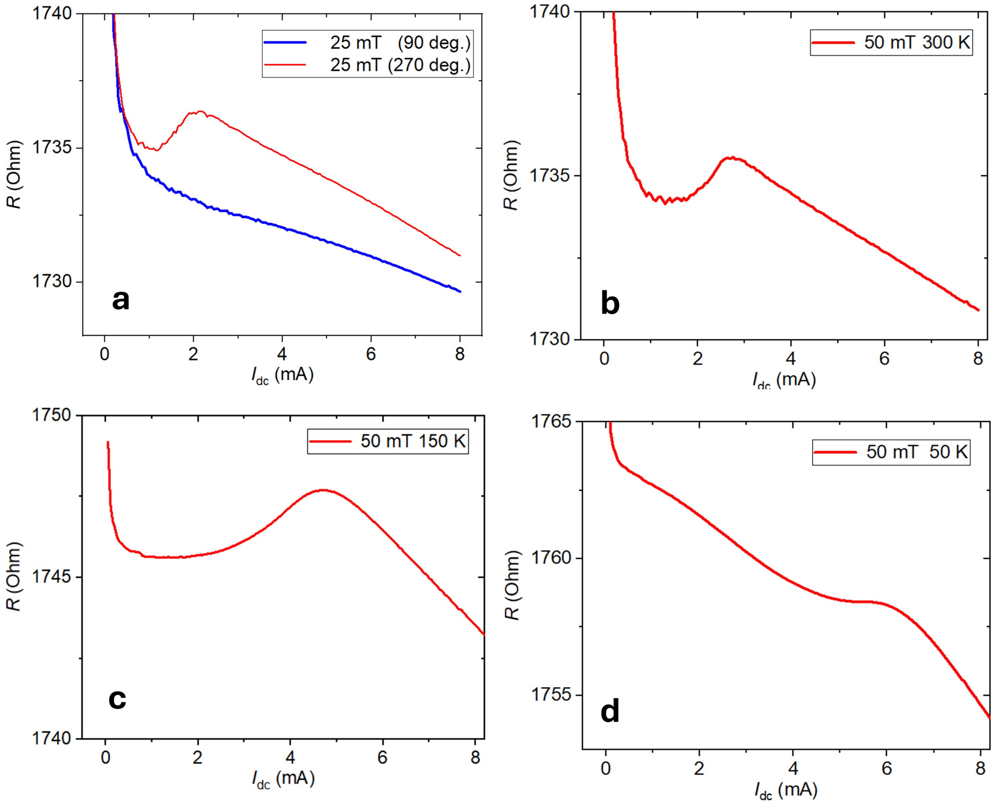

Supplementary Fig. 12. **a**, magnetoresistance measurements as a function of $I_{\rm dc}$, at room temperature, applying 25 mT along ϕ = 270 and 90 deg. respectively. **b**-**d**, magnetoresistance measurements applying 50 mT along ϕ = 270 deg. for different temperatures 300, 150 and 50 K respectively.

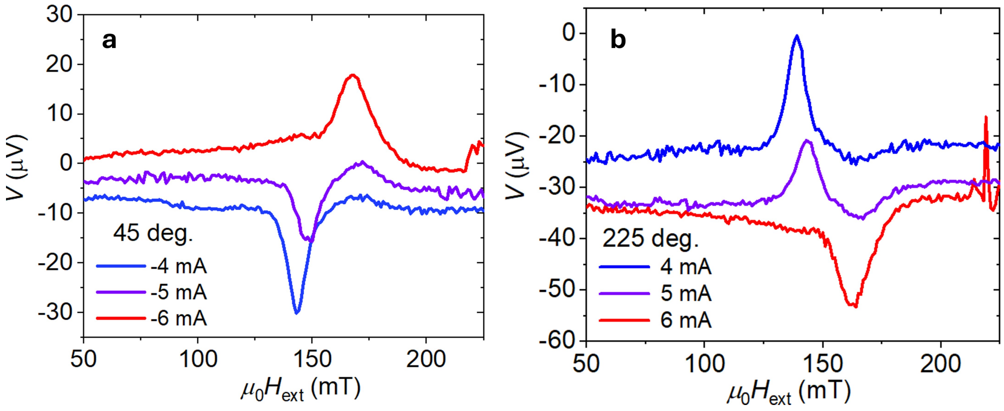

Supplementary Fig. 13. Current-induced magnetic damping control probed by ferromagnetic resonance experiments. Field-swept FMR voltages measured for **a** ϕ = 45 deg. and **b** ϕ = 225 deg. for 4 GHz while varying $I_{\rm dc}$ as specified in the figures. Voltages are offset for each figure. In both cases, clear sign switching can be observed as the magnitude of $I_{\rm dc}$ is increased for both polarities for oppositely magnetised cases to satisfy the damping compensation. These are further evidence for the time-averaging magnetisation stabilised around the direction opposite to magnetic field.

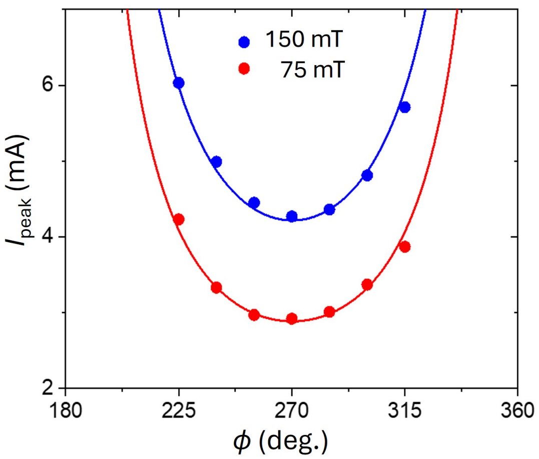

Supplementary Fig. 14. Peak current position (I_{peak}) as a function of ϕ measured at fixed field values (75 and 150 mT) with curves calculated by Eq.(1) in the main text.

References

1. Ikeda, S. *et al.* A perpendicular-anisotropy CoFeB–MgO magnetic tunnel junction. *Nat. Mater.* **9**, 721–724 (2010).
2. Yang, H. X. *et al.* First-principles investigation of the very large perpendicular magnetic anisotropy at Fe | MgO and Co | MgO interfaces. *Phys. Rev. B* **84**, 054401 (2011).
3. Ritzinger, P. & Výborný, K. Anisotropic magnetoresistance: materials, models and applications. *Royal Society Open Science* **10**, 230564.
4. Nakayama, H. *et al.* Spin Hall Magnetoresistance Induced by a Nonequilibrium Proximity Effect. *Phys. Rev. Lett.* **110**, 206601 (2013).
5. Chen, Y.-T. *et al.* Theory of spin Hall magnetoresistance. *Phys. Rev. B* **87**, 144411 (2013).
6. Avci, C. O., Mendil, J., Beach, G. S. D. & Gambardella, P. Origins of the unidirectional spin hall magnetoresistance in metallic bilayers. *Phys. Rev. Lett.* **121**, 087207 (2018).
7. Avci, C. O. *et al.* Unidirectional spin hall magnetoresistance in ferromagnet/normal metal bilayers. *Nature Physics* **11**, 570–575 (2015).
8. Borisenko, I. V., Demidov, V. E., Urazhdin, S., Rinkevich, A. B. & Demokritov, S. O. Relation between unidirectional spin hall magnetoresistance and spin current-driven magnon generation. *Applied Physics Letters* **113**, 062403 (2018).
9. Liu, G. *et al.* Magnonic unidirectional spin hall magnetoresistance in a heavy-metal–ferromagnetic-insulator bilayer. *Phys. Rev. Lett.* **127**, 207206 (2021).
10. Liu, L., Moriyama, T., Ralph, D. C. & Buhrman, R. A. Spin-Torque Ferromagnetic Resonance Induced by the Spin Hall Effect. *Phys. Rev. Lett.* **106**, 036601 (2011).
11. Garanin, D. A. Fokker-Planck and Landau-Lifshitz-Bloch equations for classical ferromagnets. *Phys. Rev. B* **55**, 3050–3057 (1997).
12. Bertotti, G., Mayergoyz, I. D. & Serpico, C. Analysis of random Landau-Lifshitz dynamics by using stochastic processes on graphs. *J. Appl. Phys.* **99**, 08F301 (2006).
13. Newhall, K. A. & Vanden-Eijnden, E. Averaged equation for energy diffusion on a graph reveals bifurcation diagram and thermally assisted reversal times in spin-torque driven nanomagnets. *J. Appl. Phys.* **113**, 184105 (2013).
14. Taniguchi, T., Utsumi, Y. & Imamura, H. Thermally activated switching rate of a nanomagnet in the presence of spin torque. *Phys. Rev. B* **88**, 214414 (2013).
15. Feller, W. Diffusion processes in one dimension. *Trans. Am. Math. Soc.* **77**, 1–31 (1954).
16. Lee, K.-M., Choi, J. W., Sok, J. & Min, B.-C. Temperature dependence of the interfacial magnetic anisotropy in W/CoFeB/MgO. *AIP Adv.* **7**, 065107 (2017).
17. Kuz'min, M. D. Shape of Temperature Dependence of Spontaneous Magnetization of Ferromagnets: Quantitative Analysis. *Phys. Rev. Lett.* **94**, 107204 (2005).
18. Callen, H. & Callen, E. The present status of the temperature dependence of magnetocrystalline anisotropy, and the power law. *J. Phys. Chem. Solids* **27**, 1271–1285 (1966).
19. Borders, W.A., Pervaiz, A.Z., Fukami, S. *et al.* Integer factorization using stochastic magnetic tunnel junctions. *Nature* **573**, 390–393 (2019).
20. Sutton, B. *et al.* Autonomous probabilistic coprocessing with petaflips per second. *IEEE Access* **8**, 157238–157252 (2020).
21. Chowdhury, S. *et al.* A Full-Stack View of Probabilistic Computing With p-Bits: Devices, Architectures, and Algorithms. *IEEE J. Explor. Solid-State Comput. Devices Circuits* **9**, 1–11 (2023).
22. Kämpfe, T. *et al.* Probabilistic Greedy Algorithm Solver Using Magnetic Tunneling Junctions for Traveling Salesman Problem,. arXiv:2501.04447.
23. Chowdhury, S., Çamsari, K. Y. & Datta, S. Emulating quantum circuits with generalized ising machines. *IEEE Access* **11**, 116944–116955 (2023).
24. Bunaiyan, S., Datta, S. & Camsari, K. Y. Heisenberg machines with programmable spin circuits. *Phys. Rev. Appl.* **22**, 014014 (2024).
25. Salakhutdinov, R., Mnih, A. & Hinton, G. Restricted boltzmann machines for collaborative filtering. In *Proceedings of the 24th international conference on Machine learning*, 791–798 (2007).

26. Bereux, N., Decelle, A., Furtlehner, C., Rosset, L. & Seoane, B. Fast training and sampling of Restricted Boltzmann Machines. In *13th International Conference on Learning Representations - ICLR 2025* (Singaour, Singapore, 2025).

27. Camsari, K. Y., Faria, R., Sutton, B. M. & Datta, S. Stochastic p-bits for invertible logic. *Phys. Rev. X* **7**, 031014 (2017).

28. Chen, H. & Murray, A. A continuous restricted boltzmann machine with a hardware- amenable learning algorithm. In Dorronsoro, J. R. (ed.) *Artificial Neural Networks — ICANN 2002*, 358–363 (Springer Berlin Heidelberg, Berlin, Heidelberg, 2002).

29. Chen, H. & Murray, A. Continuous restricted boltzmann machine with an implementable training algorithm. *IEE Proceedings - Vision, Image Signal Processing* **150**, 153–158 (2003).

30. Parra, L. & Deco, G. Continuous Boltzmann machine with rotor neurons. *Neural Networks* **8**, 375–385 (1995).

31. Harrison, R. W. Continuous restricted Boltzmann machines. *Wireless Networks* **28**, 1263 (2018).

32. Doan, K. D. *et al.* Image Generation Via Minimizing Fréchet Distance in Discriminator Feature Space. arXiv:2003.11774.

33. Wang, Z., Simoncelli, E. & Bovik, A. Multiscale structural similarity for image quality assessment. In *The Thrity-Seventh Asilomar Conference on Signals, Systems Computers, 2003*, vol. 2, 1398–1402 Vol.2 (2003).

34. Lin, J. Divergence measures based on the shannon entropy. *IEEE Transactions on Information Theory* **37**, 145–151 (1991).

35. Richardson, E. & Weiss, Y. On GANs and GMMs. In *Advances in Neural Information Processing Systems*, vol. 31 (2018).

36. Thomas, A. M., Youel, H. & Jose, S. T. Vae-qwgan: addressing mode collapse in quantum gans via autoencoding priors. *Quantum Mach. Intell.* **7**, 91 (2025).

37. Wang, Z., Bovik, A., Sheikh, H. & Simoncelli, E. Image quality assessment: from error visibility to structural similarity. *IEEE Transactions on Image Processing* **13**, 600–612 (2004).

38. Dosselmann, R. & Yang, X. A comprehensive assessment of the structural similarity index. *Signal, Image Video Processing* **5**, 81–91 (2011).
